# Mapping the conceptual structure of research on open innovation in university–industry collaborations: a bibliometric analysis

**DOI:** 10.3389/frma.2025.1693969

**Published:** 2025-11-28

**Authors:** Vladimir Alfonso Ballesteros-Ballesteros, Rodrigo Arturo Zárate-Torres

**Affiliations:** 1 Facultad de Ciencias Económicas, Administrativas y Contables, Fundación Universitaria Los Libertadores, Bogotá, DC, Colombia; 2 Colegio de Estudios Superiores de Administración (CESA), Bogotá, DC, Colombia

**Keywords:** open innovation, university–industry collaboration, higher education, academic engagement, co-word analysis

## Abstract

**Introduction:**

Open innovation has become a central mechanism for enhancing university–industry collaboration (UIC), fostering the co-development of innovative and socially responsive solutions. As organizations increasingly embrace openness and knowledge-sharing practices, understanding the evolution of open innovation in university–industry collaboration (OIUIC) is critical amid accelerating digitalization and mounting sustainability imperatives.

**Methods:**

This review maps the conceptual structure of OIUIC research from 2003 to 2024 by applying co-word analysis and social network mapping to a dataset of 2,601 articles indexed in Scopus. We extracted and standardized 5,269 unique keywords, constructed co-word networks to identify thematic clusters, and deployed network metrics to reveal patterns of scholarly collaboration and influence.

**Results:**

The analysis uncovered five dominant keyword clusters: “technology transfer,” “university–industry knowledge transfer (UIKT),” “knowledge transfer,” “academic entrepreneurship,” and “university,” which collectively define the field's conceptual architecture. Geographically, the United Kingdom leads in publication output, while *Research Policy* and *The Journal of Technology Transfer* emerge, respectively, as the most cited and the most prolific journals. Network metrics further highlight key author and institution hubs that bridge thematic communities.

**Discussion:**

By synthesizing major themes and research clusters, this review provides a comprehensive overview of the OIUIC intellectual landscape. Our findings offer critical insights for researchers and policymakers, suggesting priority areas for future inquiry, such as digital transformation, sustainability integration and cross-regional partnership models, and informing evidence-based policy development to strengthen inclusive and adaptive innovation ecosystems.

## 1 Introduction

Open innovation has emerged as a foundational concept in both academic research and organizational practice, reshaping how institutions generate, manage, and apply innovation. It is defined as a distributed process involving the strategic management of knowledge flows across organizational boundaries. In contrast to closed innovation, where firms rely exclusively on internal resources, open innovation enables organizations to enhance their innovative capacity by integrating external knowledge while expanding the scope of internal development (Chesbrough, 2003, 2006). Its effectiveness depends not only on internal capabilities but also on collaboration with external stakeholders, including suppliers, customers, universities, research institutes, consultants, and startups. The breadth and diversity of these alliances exert a direct influence on innovation outcomes (Lehtinen et al., 2019; Rossoni et al., 2024). Within this framework, university–industry collaboration constitutes a strategic space for accelerating digital transformation through open innovation (Verhoef et al., 2021), fostering the co-creation of technological solutions and scalable projects that leverage complementary capabilities across sectors. Achieving complementarity between internal and external resources, alongside capabilities such as alliance coordination and interorganizational learning, is essential for realizing its full potential (Carmona-Lavado et al., 2021).

Engagement with diverse actors facilitates the identification, acquisition, and deployment of novel ideas and technologies, thereby enhancing organizational performance. Access to external knowledge and robust network connectivity enables firms and academic institutions to capitalize on open innovation, strengthening their competitiveness in increasingly dynamic and globalized environments (Naqshbandi and Jasimuddin, 2022). In addition to improving the efficiency of product and process development, open innovation contributes to the transformation of organizational models and reinforces institutional adaptability. By channeling knowledge flows across institutional boundaries, university–industry collaborations enhance productivity, resilience, and responsiveness to technological and market shifts (Audretsch and Belitski, 2023). Moreover, open innovation expands access to external knowledge sources and promotes collaborative value creation within innovation ecosystems. These ecosystems bring together multiple stakeholders within dynamic and interconnected knowledge networks. Such configurations foster adaptability, reinforce long-term competitiveness, and support sustainable development, particularly in knowledge-intensive sectors and among micro-enterprises (Oliveira and Rua, 2025).

The transition toward a digital and sustainable society not only transforms open innovation practices but is also influenced by them. These dynamics are redefining business models and innovation ecosystems, while driving structural change within organizations. However, this evolution entails substantial challenges, particularly related to environmental impact (Robertsone and Lapina, 2023). Addressing the increasing complexity of social and economic challenges requires collaborative approaches and the integration of diverse actors to co-develop innovative solutions. In this context, emerging technologies such as big data analytics can amplify innovation capabilities and competitiveness. Nevertheless, effective implementation demands organizational readiness, strategic alignment, and efficient resource allocation to ensure impact in volatile and highly competitive environments (Sarwar et al., 2025). Open innovation not only adapts to these shifts but actively drives them by enabling new forms of collaboration and knowledge production, consolidating its role as a key enabler of sustainable and digital transformation (Bertello et al., 2024).

Open innovation and sustainability have become increasingly interconnected, as both rely on inclusive, cross-boundary collaboration and knowledge sharing to address complex societal challenges and generate socially and environmentally valuable outcomes (Kimpimäki et al., 2022). Embedding sustainability within organizational culture has been shown to strengthen innovation capabilities, enhance knowledge management systems, and improve strategic decision-making. This integration fosters institutional resilience, aligns operational practices with broader environmental and social imperatives, and supports sustained organizational performance, thereby enabling higher education institutions to navigate uncertainty and expand their societal impact (Assoratgoon and Kantabutra, 2023). Public and private stakeholders have actively supported collaborative innovation through targeted funding mechanisms and interorganizational partnerships, contributing to the development of adaptive ecosystems where sustainability and competitiveness are mutually reinforcing (Russell and Smorodinskaya, 2018). In this context, the present study examines the conceptual structure of research on Open Innovation in University–Industry Collaborations (OIUIC) from 2003 to 2024, identifying core themes, emerging trends, and influential contributors across high-impact publications, and outlining promising avenues for future inquiry.

To achieve these goals, we analyzed 2,601 documents retrieved from Scopus, widely regarded as one of the most comprehensive and reliable databases for scholarly research (Pranckute, 2021). This review contributes to the OIUIC literature by providing a systematic examination of its conceptual evolution. Prior bibliometric studies have predominantly focused on publication patterns, including authorship, institutional affiliation, and the geographical distribution of scholarly output (Randhawa et al., 2016; Gao et al., 2020; Bigliardi et al., 2021; Siriwong et al., 2024; Prasetyo et al., 2025). However, the implementation of open innovation within universities remains underexplored, limiting our understanding of its mechanisms and institutional drivers. To address this gap, this study employs co-word analysis, text mining, and social network visualization to track the conceptual development of OIUIC, identify emerging and declining themes, and assess conceptual maturity in well-developed areas. These methods enable us to map the field's intellectual structure and provide a coherent view of its historical and current trajectory.

This study adopts a mixed methods design that integrates quantitative bibliometrics with qualitative synthesis to examine the intellectual architecture of OIUIC. We analyse the field across several dimensions, including highly cited contributions, leading authors, productive countries and universities, and influential journals, to characterize its structure and evolution. This multidimensional lens helps reveal collaboration patterns, regional asymmetries in knowledge production, and the concentration of outputs within prominent research networks. In parallel, we take stock of the literature to locate underexplored themes, conceptual blind spots, and methodological bottlenecks that appear to limit cumulative progress. By triangulating metric evidence with interpretive assessment, we derive a set of targeted research directions that speak to theory development and empirical refinement. Taken together, the findings provide an integrated empirical base to inform future agendas and, in doing so, underscore the strategic role of universities as knowledge brokers and ecosystem coordinators that mobilize partnerships, reduce coordination frictions, and advance open innovation within and beyond university–industry collaboration.

Despite its expanding uptake, open innovation continues to face stubborn theoretical and empirical constraints. Inconsistent findings, ambiguous constructs, and the absence of integrative frameworks obscure effects on organizational performance and impede cumulative theory building across institutional settings (Schäper et al., 2023). Clarifying the conceptual foundations of open innovation in university–industry collaborations is therefore essential, given its interdisciplinary reach and role in knowledge creation and exchange. By reconstructing the field's intellectual trajectory, we delineate core themes, surface contested areas, and identify priority gaps that undermine coherence and comparability. Our contribution is a structured analytical framework that pairs definitional clarity with transparent, reproducible evidence to support theoretical refinement and more robust empirical accumulation. We link conceptual distinctions to operational definitions and measurement choices, indicate how modeling assumptions alter inferences, and show where consensus, contention, and ignorance remain. This positioning motivates an agenda that treats OIUIC as a plural set of mechanisms rather than a single recipe and anchors the analysis in testable propositions that travel across contexts.

We provide a balanced, comprehensive, and critical review by triangulating convergent and divergent findings across major streams, integrating reproducible co-word and network analyses with targeted full-text exemplars, and attending to regional asymmetries between the Global South and the Global North. We explicitly acknowledge database and metadata constraints and report parameter sensitivity, linking empirical patterns to core theoretical debates and actionable policy implications. The design specifies data curation, threshold choices, and robustness checks to ensure replicability, while the qualitative reading of emblematic studies clarifies mechanisms that underlie observed network structures. Together, these elements move the contribution beyond description toward evaluation, establishing where evidence is strong, conditional, or missing, and indicating where policy experiments, organizational interventions, and methodological advances are most warranted. The remainder of this article is structured as follows. Section 2 presents the theoretical foundations of OIUIC. Section 3 describes the methodological design and analytical procedures. Section 4 reports the main findings derived from the co-word and social network analysis. The final section discusses the theoretical and practical implications and proposes directions for future research.

### 1.1 Research questions

To guide our analysis of the conceptual structure and evolution of OIUIC, we address the following questions:

RQ1: What are the core conceptual themes that define the intellectual structure of research on Open Innovation in University–Industry Collaborations (OIUIC)?
RQ2: Which thematic areas within OIUIC research are established, saturated, declining, or emerging?
RQ3: How has the scholarly literature on OIUIC evolved between 2003 and 2024?
RQ4: Which articles, authors, countries, journals, and universities have exerted the greatest influence on the development of the OIUIC research field?
RQ5: What are the most promising avenues for advancing future research on OIUIC?

## 2 Theoretical background

Since its emergence, the concept of open innovation (OI) has become a central focus in academic debate and business practice, especially in an environment marked by growing globalization and the need for collaborative models that foster economic growth and competitiveness among organizations of different scales (Oliveira and Rua, 2025). In its original formulation, it is argued that valuable ideas can be generated both within and outside the organization, and their exploitation must flow bidirectionally to create value (Chesbrough, 2003, 2006). This perspective challenged the traditional paradigm of closed innovation—characterized by predominantly internal processes managed by the organizations themselves—by instead proposing a more permeable and collaborative viewpoint. Consequently, the importance of opening organizational boundaries to facilitate knowledge exchange with the external environment was highlighted (Gassmann and Enkel, 2004), integrating both internal and external sources strategically and simultaneously through multiple channels (West and Gallagher, 2006), thus shaping a dynamic and relational view of innovation.

In the years that followed, the open innovation paradigm matured, shifting its emphasis toward practical and strategic applications while underscoring the pivotal role of diverse external stakeholders in sustaining and energizing organizational innovation processes (Laursen and Salter, 2006). This expanded perspective came to include critical activities such as the acquisition, exploitation, and management of knowledge and technologies both within and beyond firm boundaries, thereby necessitating the systematic integration of dynamic capabilities (Lichtenthaler, 2008). Subsequently, open innovation was reconceptualized as a continuous, process-oriented endeavor encompassing the exploration, retention, and deployment of knowledge across traditional organizational frontiers (Lichtenthaler, 2011). Most recently, it has been characterized as a distributed system of deliberately managed knowledge flows facilitated by both pecuniary and non-pecuniary mechanisms, strategically aligned with the firm's business model (Chesbrough and Bogers, 2014) and analyzed via integrative frameworks at the micro, meso, and macro levels (Bogers et al., 2017).

Although these theoretical perspectives have significantly contributed to the field, this study adopts the original conception of open innovation, understood as a strategy that allows organizations to integrate internal capabilities with external knowledge to generate value (Chesbrough, 2006). From this standpoint, the conceptual structure of open innovation is examined within university–industry collaboration (UIC), a domain whose relevance has grown over the last decade. The academic literature has addressed this topic from various angles, analyzing antecedents and facilitators—such as organizational culture, informal relationships, human capital, and management models—and assessing its impact on institutional performance, with findings ranging from positive to neutral or negative, depending on context and existing complementary capabilities. In the face of this fragmented panorama, this study applies co-word analysis, an effective tool for identifying thematic lines, recognizing key concepts, and clarifying connections in emerging or dispersed research fields. This approach clarifies how open innovation unfolds across varied organizational environments.

OI has consolidated its position as a fundamental paradigm that propels the strategic transfer and management of knowledge through organizational openness to external actors. This approach enriches interactions, creates business and technological opportunities, and fosters collaborative dynamics that enhance innovation, reinforce competitiveness, and stimulate economic growth (Flamini et al., 2022). In this context, UIC plays a pivotal role by strategically connecting scientific, academic, and technological capacities developed in universities with the applied knowledge and specific needs of the business sector, thereby facilitating the creation of innovations with substantial economic, technological, and social impacts (Rossi et al., 2024). These alliances promote concrete spaces for co-creation, such as technology transfer offices, joint research centers, incubators, and science parks, where both parties share resources, risks, and benefits, expediting the development and successful deployment of tailored technological innovations (Hailu, 2024). This synergy underscores how OI contributes to real-world innovation outcomes and fosters cross-disciplinary collaborations.

University–industry collaboration (UIC) serves various strategic purposes, notably the strengthening of organizational capacities to carry out joint initiatives. Beyond enabling sustainable links, these alliances encourage the building of structural, relational, and cognitive social capital, thereby laying a foundation for broadening the impacts generated beyond the initial relationships among the participating actors (Sa'a and Asplund, 2025). To achieve this, formal agreements, clear policies, and mechanisms that cultivate mutual trust and institutional commitment are essential. Likewise, it is indispensable to invest in human capital development through specialized training, shared learning, and mentoring programs. These conditions help establish solid partnerships that facilitate specific outcomes such as the formation of highly qualified talent, execution of high-impact collaborative research, and the establishment of transparent, equitable agreements (Tucker et al., 2024). Complementarily, the collaborative process often advances in progressive stages—embryonic, initiation, involvement, and consolidation—each focused on deepening and sustaining long-term collaboration (O'Dwyer et al., 2023). Collectively, these measures fortify the collaborative base for sustainable innovation.

University–industry collaboration (UIC) is increasingly supported by multi-level governance frameworks that strategically align institutional capabilities with regional priorities, thereby fostering synergistic interactions and coordinated knowledge transfer processes (Albers et al., 2016). These frameworks promote mutual trust and institutional commitment, while also enabling equitable partnerships aimed at delivering shared innovation outcomes (Clauss et al., 2024). By reducing organizational misalignments, they improve the interface between academic research and industry needs through mechanisms that facilitate technology commercialization and foster more seamless engagement (Tolin and Piccaluga, 2025). In this evolving landscape, alternative supervision models—particularly co-supervision with non-academic professionals—have gained prominence. These actors, often embedded in industry or applied research settings, contribute contextualized expertise and practical insights that enhance the societal relevance of doctoral education (McAlpine et al., 2020). Their involvement ranges from targeted advisory roles to deep collaborative engagement, including joint research, experiential learning, and co-authored outputs (Sun and Turner, 2023). Together, these practices reinforce the integrative function of UIC and amplify its contribution to the development of resilient, innovation-driven ecosystems.

Beyond immediate benefits, UIC produces effects that transcend educational and technological spheres, extending to social, economic, and institutional dimensions, with indirect benefits that accrue to communities, regions, and actors not directly involved (Cohen et al., 2024). In particular, collaborative networks emerging within regional innovation systems enhance local capacities for generating and applying knowledge, facilitate technology transfer, and support sustainable economic growth by aligning multiple stakeholders around common goals (Luongo et al., 2023). Such dynamics also drive the formation of science- and technology-based ventures, promote the creation of skilled jobs, and bolster competitiveness across production ecosystems (Schiuma and Carlucci, 2018). From an academic perspective, ongoing engagement with industry promotes up-to-date teaching content, provides curricular flexibility, and strengthens the link between higher education and real innovation processes (Lundberg and Öberg, 2021). Collectively, these mechanisms enrich university education and support sustainable and inclusive territorial development pathways (Hojeij, 2024). By enriching educational pathways and fostering inclusive growth, UIC exemplifies the far-reaching significance of collaborative models (Ahmed et al., 2022).

Within the OIUIC framework, three collaboration modes are commonly identified: inbound licensing, outbound licensing, and co-development (Gustina et al., 2024). Outbound licensing, via research contracts, consultancy, and technical services, dominates because its formal contractual arrangements and rapid execution ensure efficient knowledge transfer from universities to industry (Ankrah and Omar, 2015). Inbound licensing, whereby universities adopt external knowledge or technologies, is less prevalent; this reflects concerns about knowledge spillover, low inter-organizational trust, and the difficulty of managing intellectual property when tacit knowledge predominates and legal frameworks are weak (Ayerbe et al., 2024). Co-development involves joint R&D initiatives in which partners share resources, expertise, and risks, thereby fostering strategic, long-term partnerships aligned with mutual objectives (Mascarenhas et al., 2024). Together, these modes enable collaborators to integrate complementary capabilities, enhance innovation outcomes, shorten R&D timelines, reduce costs, and improve responsiveness to complex and dynamic environments (Awasthy et al., 2020).

Likewise, two essential practices arise within this collaborative framework to significantly aid in achieving positive outcomes: networking and strategic alliances (Randhawa et al., 2016). Networking involves creating, managing, and maintaining dynamic networks that bring together academic, industrial, and community actors through multiple communication channels. This fosters a continuous exchange of information, opens up collaboration opportunities, and enables agile adaptation to shifts in socioeconomic conditions (Gao et al., 2020). To maintain effective networking, technological tools such as databases, digital platforms, electronic bulletins, and connectivity teams are used, expanding both the reach and the efficiency of interactions (Bigliardi et al., 2021). Strategic alliances, on the other hand, are more formalized and structured relationships, based on enduring commitments and clearly defined procedures. These alliances promote coordination, collective learning, and the co-creation of shared value, thus effectively addressing complex challenges (Cassiman and Veugelers, 2006). In doing so, stakeholders benefit from deepened engagement and broadened opportunities for innovation.

In conclusion, open innovation and university–industry collaboration have become increasingly relevant for driving effective co-creation processes, optimizing resource utilization, and enhancing competitiveness within an environment shaped by rapid knowledge evolution. Strategies built upon openness, social capital development, and formal cooperation mechanisms enable organizations to overcome traditional barriers, facilitating the efficient transfer of ideas, technologies, and innovative practices. These collaborative dynamics produce mutual benefits for universities, industry, and society at large, bridging the gap between advanced knowledge generation and its practical application within productive sectors. In contemporary contexts, the adoption of collaborative frameworks has transcended merely gaining competitive advantages; rather, it is now an essential requirement for confronting the intensified challenges presented by an increasingly globalized, competitive, and constantly changing landscape. Moreover, the establishment of enduring partnerships and effective governance structures significantly contributes to ultimately securing concrete and sustainable outcomes, ensuring long-term success and meaningful impacts from these multifaceted collaborative endeavors.

## 3 Research methodology

### 3.1 Definition of the research field

This study conducts a bibliometric analysis of the field of open innovation in university–industry collaborations (OIUIC), combining co-word techniques and social network analysis. This methodological strategy enables the examination of the field's conceptual structure, the identification of thematic clusters, the mapping of semantic relationships among key terms, and the detection of collaboration patterns among authors and institutions. Through this approach, the study delineates the field's scope, highlights major lines of development, and traces its intellectual evolution, thereby contributing to a more systematic and articulated understanding of its current configuration.

### 3.2 Research design and analytical framework

This study adopts a quantitative bibliometric design for 2003 to 2024. Data were retrieved from a leading citation index and curated prior to analysis. Using standard bibliometric software, including VOSviewer, we combine co-word analysis and social network analysis to examine the conceptual structure of research on OIUIC, delineate core and emerging themes, and map influential contributors across authors, institutions, and sources. Descriptive indicators of output, impact, and geographic distribution complement the network results to characterize scientific production and its evolution across countries and journals. Taken together, these procedures provide an objective synthesis of the field's intellectual organization and thematic dynamics, offering a rigorous and comprehensive view of the research landscape and guidance for future inquiry. Sections 3.2–3.5 detail the co-word procedure, the social network approach, the VOSviewer implementation, and the definition of the research field.

### 3.3 Co-word analysis

Co-word analysis is a bibliometric technique employed to identify association patterns among key terms present in the scientific literature (Callon et al., 1986). Since its introduction, it has been widely adopted to map the intellectual structure of diverse research fields, such as brand equity (Rojas Lamorena et al., 2022), university–industry knowledge transfer (UIKT) (Ballesteros Ballesteros and Zárate Torres, 2025), ecopreneurship (Guleria and Kaur, 2021), neuromarketing and consumer behavior (Kajla et al., 2024), and artificial intelligence and big data in information systems (Dwivedi et al., 2023), among others. This method relies on statistically identifying the co-occurrence of key terms, uncovering conceptual connections among specific lexical components, thereby facilitating the detection of thematic cores, emerging research strands, and evolving dynamics within the studied area (Zhu and Zhang, 2020). A fundamental advantage is its ability to infer the conceptual structure of a research field without requiring exhaustive analysis of each document's full content, thus enhancing efficiency and ensuring greater analytical objectivity (Öztürk et al., 2024). As a result, co-word analysis has become a valuable tool for understanding the development of scientific domains and anticipating future research trajectories.

Co-word analysis operates on the assumption that authors deliberately select keywords to represent the core concepts investigated in their publications. Keyword co-occurrence analysis identifies frequently associated pairs of terms in the literature, which serve as indicators of latent conceptual relationships. By mapping these co-occurrences, the method reveals the intellectual structure of a research field and uncovers dominant themes and coherent research clusters (Farea et al., 2024). In addition to identifying internal associations, this technique also detects conceptual bridges between otherwise disconnected areas of knowledge by capturing peripheral or interdisciplinary terms that display significant co-occurrence patterns across the scientific discourse (Giannakos et al., 2020). As an integrative bibliometric approach, co-word analysis enables the systematic exploration of keyword relationships, supporting the identification of influential concepts, the delineation of thematic configurations, and the detection of emerging research fronts. By producing visual representations of a field's intellectual architecture and historical progression, co-word analysis offers a robust empirical foundation for understanding conceptual evolution and guiding future scholarly inquiry and research agenda setting (Phan Tan, 2021).

Additionally, co-word analysis allows the construction of taxonomies and theoretical frameworks by clustering key terms into thematic groups, which facilitates systematic knowledge organization and the development of integrative theoretical perspectives. Such structures are particularly valuable for navigating extensive and complex literature bodies, as they clearly identify central concepts, peripheral topics, and significant gaps within the analyzed field (Klarin, 2024). Moreover, this technique enables tracking transformations within subfields, revealing emerging conceptual relationships, and highlighting patterns characterized by thematic ambivalence, complementarity, or independence, thus reflecting continuously evolving intellectual dynamics (Scharp, 2021). Overall, co-word analysis not only analytically represents the current state of knowledge but also serves as a prospective tool to guide the formulation of future research agendas by uncovering novel conceptual connections and topics with potential for further development (Corrales Garay et al., 2019).

### 3.4 Social network analysis

Social network analysis (SNA) is a powerful framework for examining scholarly ecosystems, as it reveals interdependencies among researchers, publications, and institutions by constructing co-authorship and citation networks (Freeman, 2004; Tabassum et al., 2018). Rather than treating academic outputs as isolated artifacts, SNA positions them as nodes within complex networks of collaboration and influence, enabling the detection of relational patterns and knowledge flows that drive scientific development (Van Der Valk and Gijsbers, 2010). These networks can be analyzed longitudinally to capture the evolution of research communities over time. Network visualizations map alliances among authors and institutions and trace the distribution of intellectual capital across research communities. By applying metrics such as centrality, efficiency, authority, and hub scores, SNA identifies leading contributors and elucidates the institutional configurations that shape academic performance. This approach also enables the examination of interdisciplinary linkages and thematic clusters. Moreover, SNA supports the integration of bibliometric and altmetric data to provide a multidimensional perspective on research impact (Xu and Chang, 2020).

One of the most salient applications of social network analysis (SNA) in scientific research lies in the examination of co-authorship networks, where each collaborative tie among researchers constitutes a relational link that reflects the structural configuration of an academic field. This approach enables the identification of influential actors, the mapping of interaction patterns, and the analysis of how scientific output is collectively produced and sustained over time (Acedo et al., 2006). Beyond co-authorship, SNA offers a comprehensive framework for identifying structurally central nodes within complex knowledge networks, providing insights into the circulation of ideas, the diffusion of information, and the formation of epistemic communities (Litterio et al., 2017). By capturing indirect ties and latent structures, it enables the identification of cohesive subgroups and the reconstruction of thematic trajectories. Furthermore, by uncovering the composition and interconnections among research clusters, SNA facilitates the identification of established and emerging areas, the visualization of knowledge structures, and the mapping of thematic evolution. In business and management studies, it supports the analysis of shifting scholarly trends and provides strategic insights for guiding future research agendas (Anugerah et al., 2022).

SNA also provides powerful visual representations of co-authorship networks, offering an intuitive understanding of relationships among researchers, topics, and knowledge domains. These visualizations reveal the underlying collaborative structure and flows of expertise across topics, supporting the analysis of the dynamic evolution of interdisciplinary scientific production (Schäfermeier et al., 2023). In such graphs, nodes represent scientific actors, while edges reflect collaborative ties, enabling the identification of both established structures and peripheral or unexpected connections between research subfields (Ullah et al., 2022). Beyond its analytical value, co-authorship networks constitute a strategic asset for academic knowledge management, as they allow institutions to identify key nodes, reliable ties, and influential publication trajectories. This structural insight supports decisions related to scientific collaboration, resource allocation, and the formulation of research agendas, ultimately enhancing institutional impact and productivity (Li et al., 2013). Integrating SNA thus offers a deeper understanding of how scientific knowledge is generated and disseminated.

### 3.5 VOSviewer

VOSviewer has become a cornerstone in bibliometric research, offering advanced functionalities for constructing and visualizing scientific networks based on co-authorship, co-citation, and co-occurrence relationships (Bukar et al., 2023, 2025). Its intuitive interface and high-resolution graphical outputs have established it as a reference tool for structural analyses of academic literature. By linking researchers, publications, and journals through multiple relational indicators, VOSviewer produces detailed maps that reveal latent thematic associations and patterns of collaboration across disciplinary boundaries (Xie et al., 2025). A key strength of bibliometric analysis lies in its ability to integrate diverse relational techniques, such as direct citation, bibliographic coupling, co-citation, and co-authorship. Each technique provides a distinct analytical perspective that contributes to a multidimensional understanding of a field's intellectual structure and scholarly dynamics (Fagang et al., 2025). Specifically, VOSviewer's co-citation analysis identifies clusters of frequently cited documents, uncovering cohesive scholarly communities, while author co-citation density maps highlight central contributors. Additionally, bibliographic coupling visualizes clusters of journals and publications that share references, grouping them by thematic proximity and exposing major research areas and emerging trends that trace the evolution and consolidation of knowledge within scientific ecosystems (Benatiya Andaloussi, 2024).

Beyond its established capability for network visualization, VOSviewer incorporates a text analysis module applied to titles and abstracts, significantly enhancing bibliometric studies from a conceptual perspective (Lim et al., 2024). Utilizing extensive data volumes, this tool extracts keywords and generates co-occurrence networks to identify frequently co-occurring terms, thereby aiding in the delineation of thematic clusters, semantic analysis, and detection of emerging trends in dynamic fields (Haba et al., 2023). This procedure is essential for tracing the conceptual evolution within specific areas, pinpointing emerging research topics, and identifying shifts in theoretical frameworks. By interpreting these patterns, researchers can track the increasing prominence of certain topics, articulate interconnections between subfields, and observe changes in scientific agendas over time (Amiruddin et al., 2025). Thus, VOSviewer serves as an analytical platform that effectively reveals conceptual gaps and interdisciplinary integration opportunities.

Overall, VOSviewer has become an essential tool in bibliometric analysis by enabling interactive visualizations that explore thematic structures, collaboration networks, and trends in scientific production (Amiruddin et al., 2025). Its capability to construct and represent networks based on various relationships, coupled with advanced text-mining functions, positions this platform strategically to investigate the creation, linkage, and dissemination of scientific knowledge. Additionally, its intuitive interface facilitates adoption by researchers with diverse levels of bibliometric expertise, contributing to its widespread use within the academic community. This combination of technical versatility, user accessibility, and analytical capacity makes VOSviewer instrumental in strategic knowledge mapping, the identification of emerging trends, and the formulation of research agendas aligned with the actual dynamics of scientific production across different fields.

### 3.6 Procedural transparency and replicability

**Figure 1:**
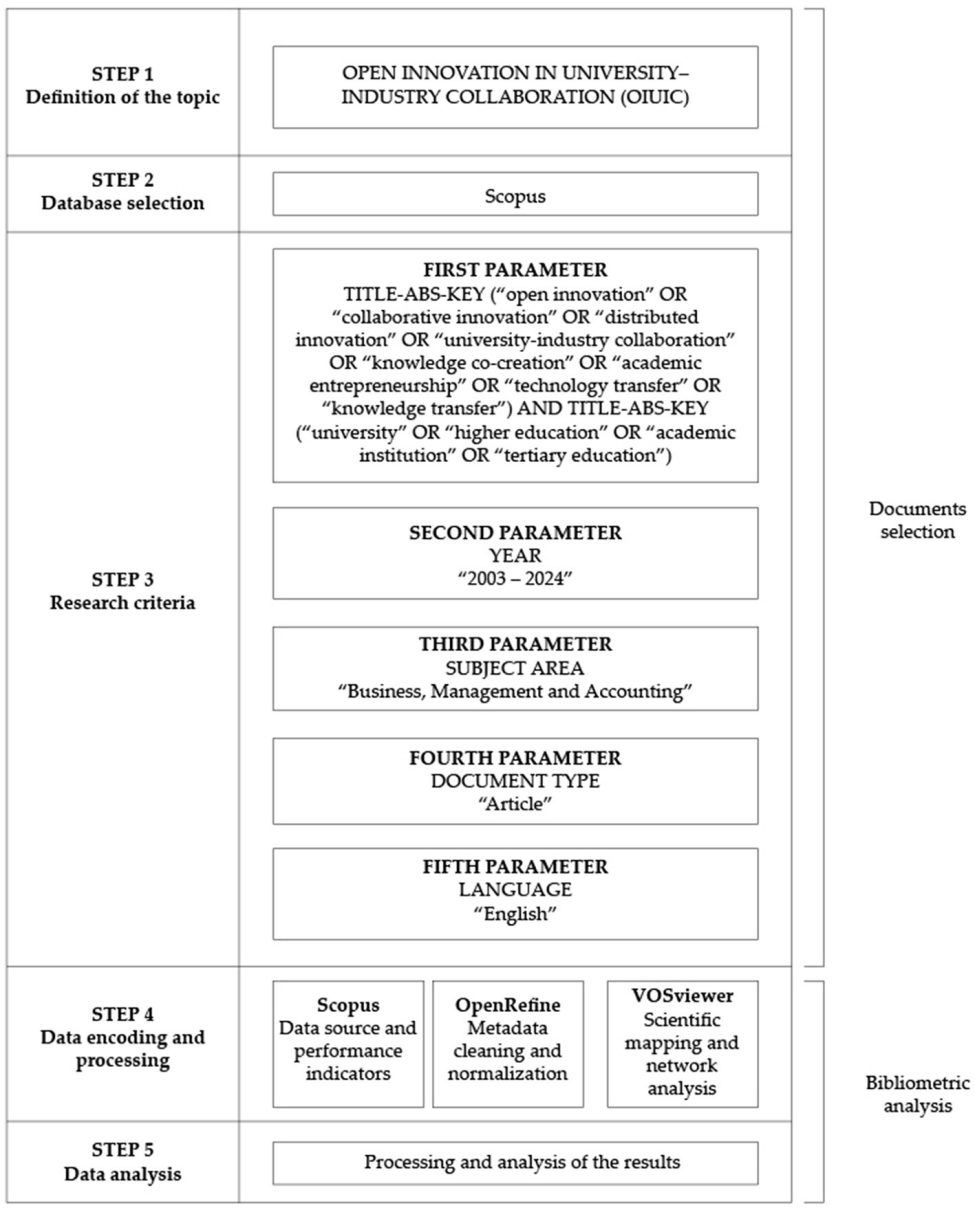
Search protocol and flow diagram adapted from Vergura et al. (2023).

Details of the data source, snapshot, inclusion criteria, deduplication, exported variables, and keyword cleaning are reported in Figure 1. Below we document the analytical pipeline and the exact thresholds used so the results can be reproduced without revisiting the data-collection narrative.

• Step 1: Keyword universe and reduction (co-word)
Starting from the cleaned keyword field shown in Figure 1, we constructed a co-occurrence matrix in VOSviewer. A minimum occurrence threshold of 15 was applied, yielding 79 recurrent terms for conceptual mapping. Clusters were interpreted using term weights, intra-cluster links, and representative documents. For clarity, we used a minimum cluster size of 10 and a standard clustering resolution.
• Step 2: Co-authorship networks
Collaboration graphs were built at author, organization, and country levels. For interpretability, inclusion filters were set as follows: authors with ≥3 documents and ≥10 citations (resulting in 330 authors) and countries with ≥10 documents and ≥20 citations (resulting in 49 countries). Isolates were removed only from figures for readability; counts are reported with the descriptive results.
• Step 3: Co-citation and venue structure
To characterize the intellectual base, cited-reference co-citation maps were produced with a minimum of 35 citations, resulting in 93 cited documents. For venue structure, source (journal) co-citation maps were also generated; the citation threshold was adjusted to obtain an interpretable network size. All maps were created in VOSviewer with its standard settings unless otherwise stated in the figure captions.
• Step 4: Visualization and reporting
Analyses were conducted in VOSviewer using the default layout. Node size reflects the relevant weight (occurrences, documents, or citations), and link thickness reflects relatedness. The final thresholds applied to each map are stated in the corresponding figure captions.

Table 1 consolidates the thresholds and settings used to generate each network displayed in Figures 3–9. For every map we report the analysis type, unit of analysis, inclusion rule(s), and the resulting number of nodes; isolates were removed from the visualizations only. Thresholds were tuned to balance coverage and interpretability (targeting ~100–200 nodes where applicable), enabling straightforward replication from the same Scopus snapshot.

**Table 1:** Summary of methodological choices.

**Map/figure**	**Analysis**	**Unit**	**Inclusion threshold(s)**	**Resulting nodes**	**Notes**
Co-word (Figure 3)	Co-occurrence	Keywords	Occurrences ≥ 15; minimum cluster size = 10	79	Standard clustering resolution used
Co-citation (Figure 5)	Co-citation	Cited documents	Citations ≥ 35	93	Intellectual base map
Co-authorship (authors) (Figure 6)	Co-authorship	Authors	Documents ≥ 3 and Citations ≥ 10	330	Isolates removed only in figures
Co-authorship (countries) (Figure 7)	Co-authorship	Countries	Documents ≥ 10 and Citations ≥ 20	49	Isolates removed only in figures
Co-citation (sources) (Figure 8)	Co-citation	Sources (journals)	Citation threshold adjusted for interpretable size	151	Dense structure with strong link strengths
Co-authorship (organizations) (Figure 9)	Co-authorship	Organizations	Thresholds as applied in the figure	148	Graph sized to 100–200 nodes for interpretability; isolates removed only in figures.

### 3.7 Data collection, refinement, and standardization

On February 13, 2025, bibliographic metadata related to research on open innovation in university–industry collaborations (OIUIC) were retrieved from the Scopus database and exported in CSV format. The dataset included essential variables for conducting a co-word analysis, such as author names, document titles, keywords, institutional affiliations, publication years, and citation counts. The search strategy combined terms associated with open innovation, university–industry collaboration, knowledge co-creation, academic entrepreneurship, technology transfer, and knowledge transfer, alongside descriptors referring to universities and other higher education institutions. This process yielded an initial set of 12,281 records. To refine the dataset, inclusion criteria were applied to retain only journal articles published between 2003 and 2024, written in English, and indexed under the Business, Management and Accounting subject area. After removing duplicates, the final dataset consisted of 2,601 documents.

To ensure analytical consistency, a keyword standardization process was implemented to address grammatical, orthographic, and semantic variations, such as university vs. universities and patent vs. patents. This procedure was carried out using OpenRefine (version 3.8.7), an open-source tool for data cleaning and transformation. The CSV file was imported into OpenRefine, and the keyword column was examined for inconsistencies. Using the Text Facet function, similar terms were identified and grouped to construct a thesaurus that consolidated variants into standardized forms. For instance, terms such as “*University spinoffs,” “Academic spinoff,” “University spin-offs,” “Spinoffs,”* and “*university spin-off”* were harmonized under the unified label *university spin-offs*. This choice reflects its frequency in the academic literature, semantic clarity, and syntactic consistency. The thesaurus was then applied across the dataset to ensure terminological coherence. This process enhanced the reliability of the co-word analysis and contributed to a more accurate mapping of the field's conceptual structure.

Figure 1 provides a detailed overview of the data collection, refinement, and standardization procedures.

## 4 Results

**Figure 2:**
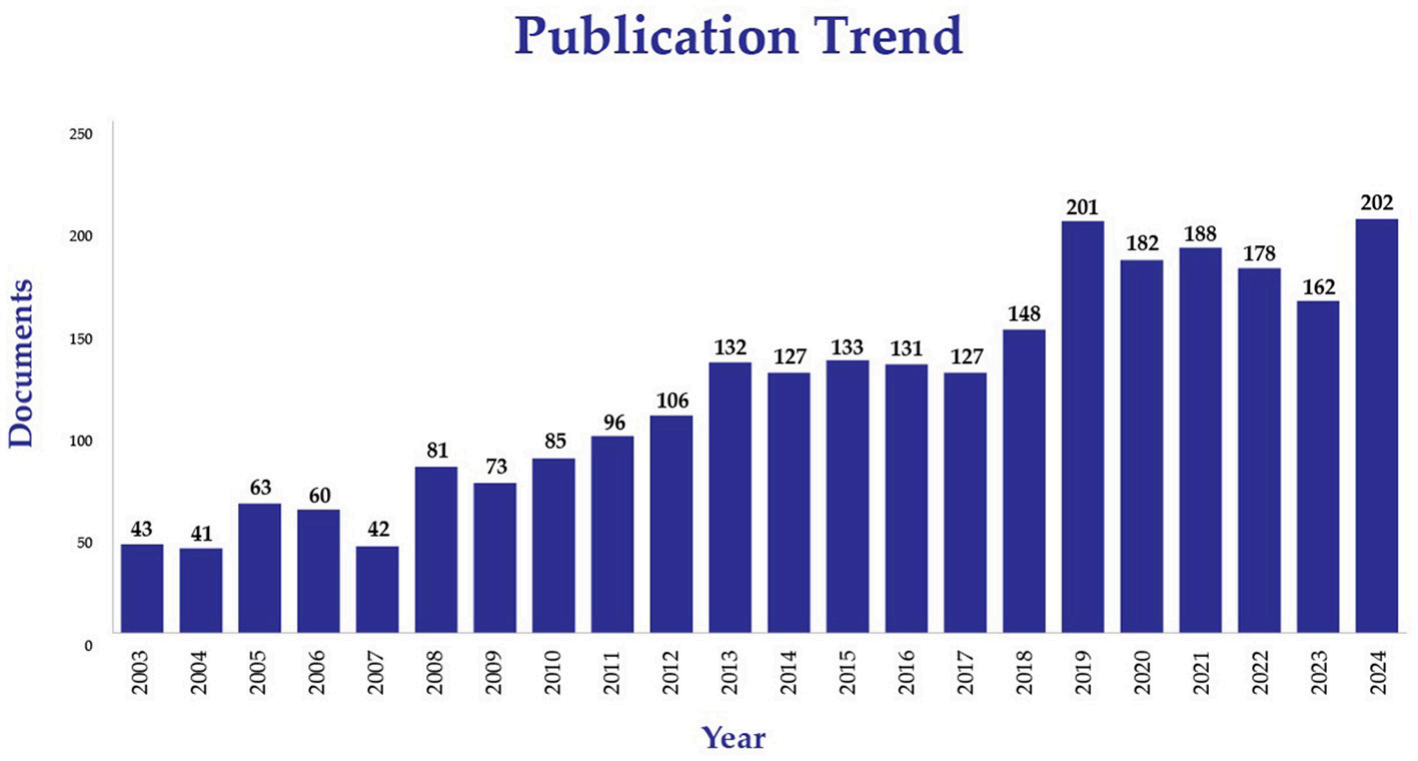
Publication trend in OIUIC between 2003 and 2024.

The evolution of annual publication output between 2003 and 2024 reflects the growing academic interest in open innovation in university–industry collaborations (OIUIC). Over this period, the number of scholarly articles addressing the topic increased steadily, suggesting its consolidation as a relevant and dynamic area within the broader innovation studies literature. As shown in Figure 2, the dataset comprises 2,601 documents, with a marked upward trend in publication volume across the two decades. The year 2024 stands out as the most prolific, with a total of 202 articles, underscoring the intensifying focus of the academic community on the conceptual and empirical dimensions of OIUIC.

### 4.1 Keyword frequency and trends

Co-word analysis examines the frequency with which specific keywords co-occur across scientific publications, revealing thematic patterns and levels of conceptual concentration in academic output (Sampagnaro, 2023). This technique enables the identification of semantic relationships among terms and the formation of clusters that reflect the conceptual structure of a given research domain (Dwivedi et al., 2023). When applied to bibliometric studies, co-word analysis supports the detection of latent patterns and the organized representation of knowledge by mapping the intellectual linkages that shape a discipline (Rejeb et al., 2025). Through this approach, researchers can uncover thematic connections, trace the conceptual evolution of a field, and identify emerging lines of inquiry. Additionally, co-word analysis facilitates the generation of thematic maps that capture the intellectual dynamics of a research area and inform future research agendas (Arokiasamy et al., 2024). It also enhances information retrieval and enables the visualization of knowledge networks and emerging thematic communities within the academic ecosystem (Aparicio et al., 2024), consolidating its value as a strategic research tool.

Building on this foundation, the dataset was processed and standardized using OpenRefine, ensuring terminological consistency and the accuracy of the keywords included in the co-word analysis. The initial corpus comprised 5,269 terms extracted from academic publications dated between 2003 and 2024. To reduce analytical complexity and focus on conceptually significant content, a minimum frequency threshold of 15 occurrences was applied. This filtering step yielded a refined subset of 79 recurrent and thematically relevant keywords, representative of the main research streams in the field of open innovation in university–industry collaborations (OIUIC). Table 2 presents the 20 most frequent keywords, providing an empirical overview of dominant thematic trends. This preliminary analysis serves as a foundation for constructing the co-word network on which the subsequent structural examination of the field's conceptual organization is based.

**Table 2:** Top 20 keywords.

**Keyword**	**Frequency**	**Keyword**	**Frequency**
Technology transfer	547	Commercialization	88
UIKT	465	Patents	82
Knowledge transfer	322	R&D	74
Academic entrepreneurship	309	Knowledge management	61
University	276	Intellectual property	60
Innovation	250	SMEs	56
University spin-offs	223	University technology transfer	55
Entrepreneurial university	141	Collaboration	52
Entrepreneurship	130	Startups	51
Higher education	100	Triple helix	47

**Figure 3:**
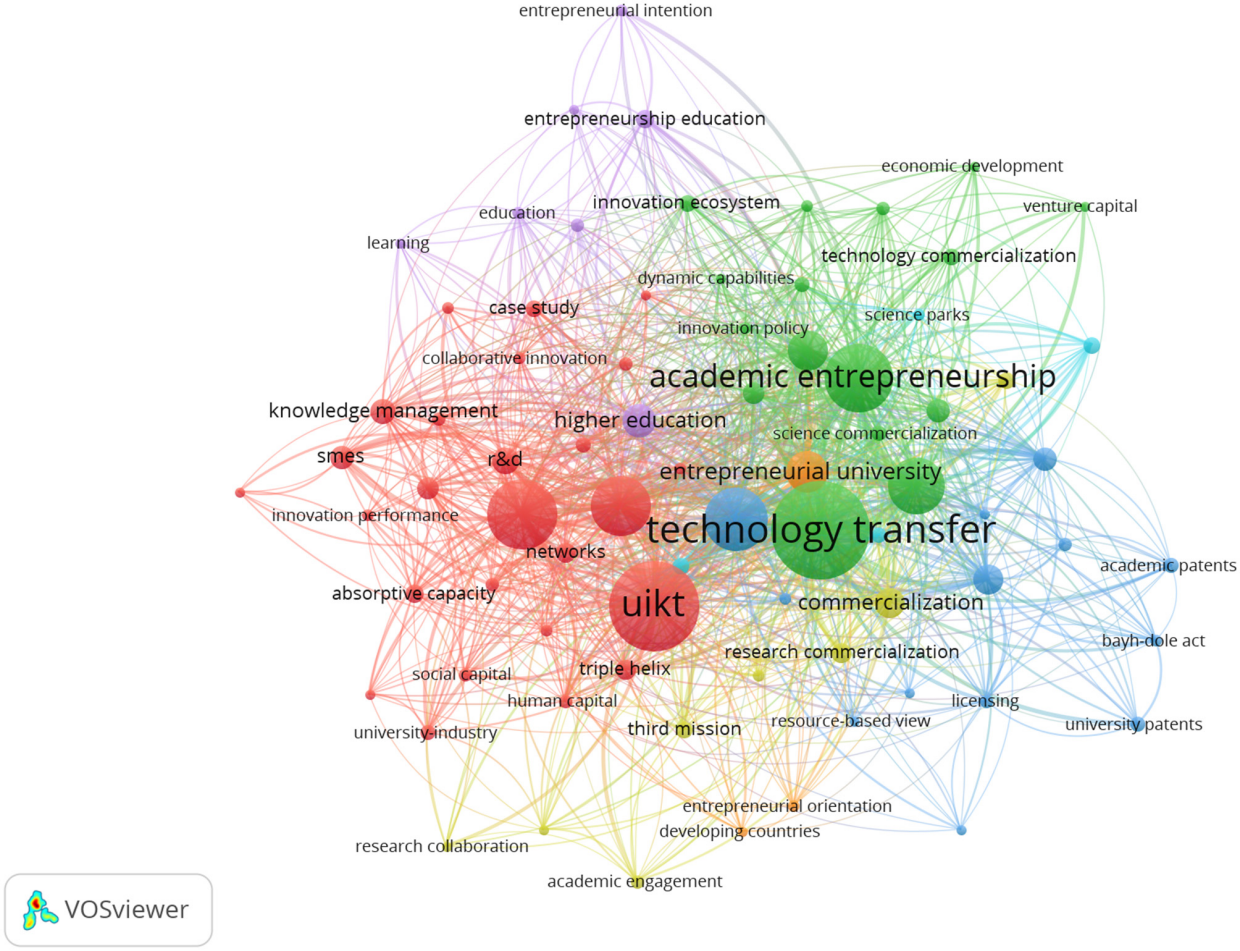
Co-occurrence network of keywords (2003–2024).

Co-occurrence analysis was applied to identify the conceptual structure of research on Open Innovation in University–Industry Collaborations (OIUIC) through the mapping of relationships among keywords extracted from scientific publications. This technique enabled the examination of how specific terms consistently appear together, revealing thematic affinities, conceptual proximity, and the emergence of specialized subfields within the domain. The resulting network, depicted in Figure 3, comprises seven thematic clusters that operate as conceptual cores around which the field is organized. Each cluster groups interrelated terms that reflect both the persistence of classical approaches—centered on formal technology transfer—and the integration of contemporary perspectives that emphasize the interaction between universities and a variety of social, economic, and territorial actors. This thematic configuration highlights the coexistence of distinct conceptual traditions and provides a robust empirical foundation for analyzing the intellectual development of the field, as well as its degree of diversification and specialization.

Cluster 1 groups concepts related to collaborative innovation, absorptive capacity, knowledge transfer, and networks. This thematic area explores how universities interact with firms and other actors to mobilize knowledge within the context of open innovation in university–industry collaborations (OIUIC). At the core of this cluster is UIKT (University–Industry Knowledge Transfer), a central construct that reflects the interplay between organizational capabilities, such as R&D, knowledge management, and SMEs, and systemic structures like triple helix arrangements. Cluster 2 focuses on the entrepreneurial transformation of academic institutions, integrating terms such as academic entrepreneurship, entrepreneurial university, entrepreneurship, and university spin-offs. These terms reflect how universities contribute to regional innovation systems by fostering entrepreneurial activity. Cluster 3 addresses formal mechanisms of knowledge appropriation, including technology transfer, university technology transfer, intellectual property, licensing, and patents, emphasizing the institutional frameworks that support commercialization in OIUIC settings.

Cluster 4 revolves around the notion of academic engagement and its articulation with the third mission, including terms such as research collaboration, knowledge commercialization, and university–industry partnerships. This cluster captures a growing strand of literature that examines how universities engage in co-productive relationships with external stakeholders. Cluster 5, oriented toward entrepreneurship education, incorporates terms like higher education, student entrepreneurship, and social networks, underscoring the role of universities in building entrepreneurial competencies. Cluster 6 links open innovation to regional development, emphasizing infrastructures such as science parks and models like regional innovation systems that support territorially grounded collaboration. Finally, Cluster 7 introduces the specific challenges faced by developing countries, where limited institutional and technological capacity demands renewed attention to entrepreneurial orientation. Together, these clusters delineate the conceptual structure of OIUIC research, reflecting distinct but interrelated lines of inquiry.

The co-word network and associated keyword structure indicate a tightly connected conceptual field. Rather than following a single linear pathway, research on OIUIC evolves through the convergence of complementary perspectives. The prominence of university–industry knowledge transfer (UIKT), technology transfer, and academic entrepreneurship is consistent with an integrative role across clusters, as reflected in above-median degree and betweenness values and multiple cross-cluster links in the keyword map (see Figure 3; Table 2). Universities appear as hybrid institutions that pursue scientific advancement, knowledge valorisation, and social impact within a systemic view of innovation. The thematic organization suggests that internal policies such as R&D strategies, technology transfer offices, and organizational culture interact with enabling conditions that include public policy frameworks, collaborative networks, and territorial dynamics (Jutidharabongse et al., 2024). Taken together, the analysis points to increasing diversification, the coexistence of commercialization and engagement logics, and the gradual emergence of inclusive innovation models adapted to varied institutional contexts.

**Figure 4:**
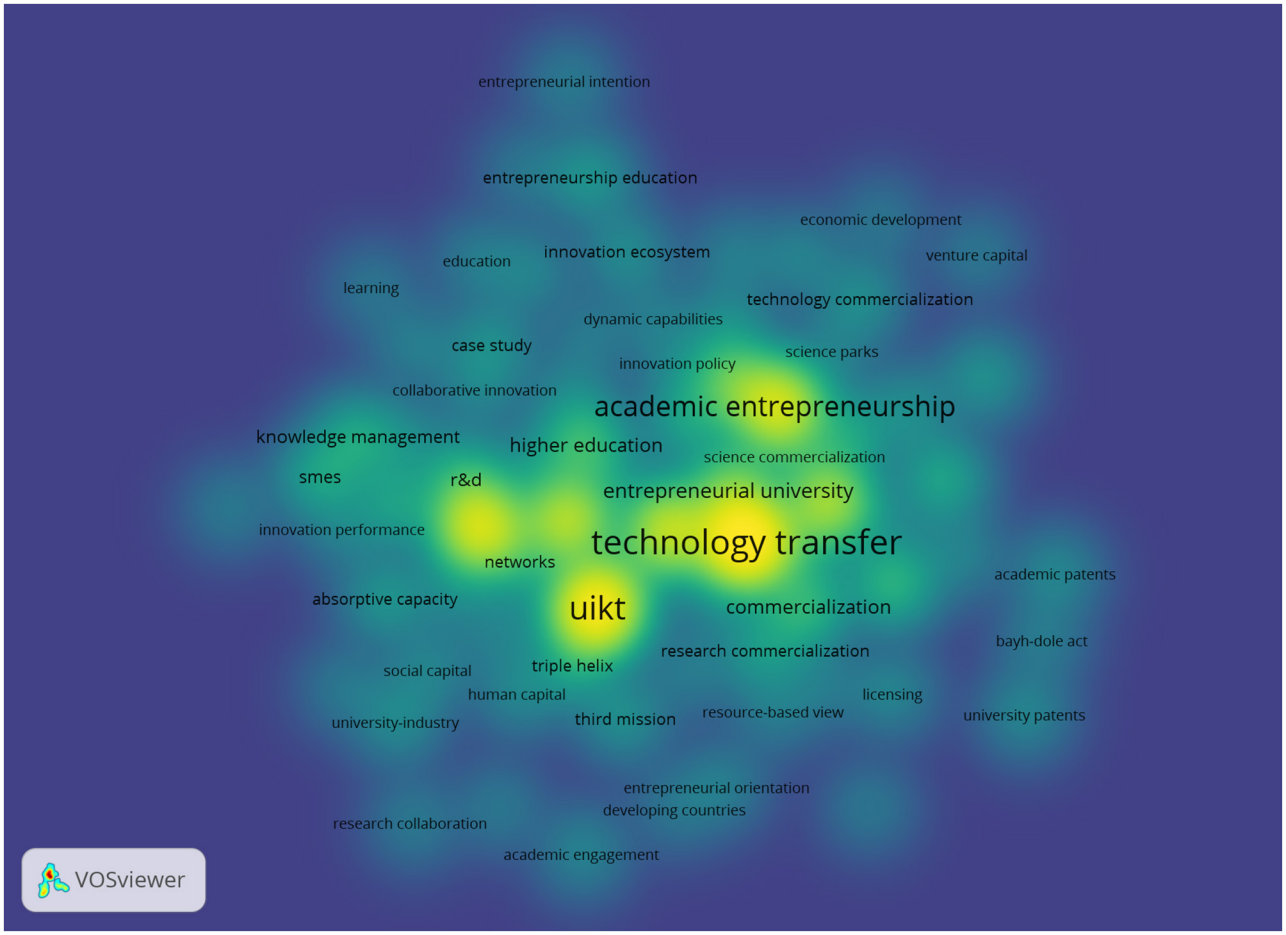
Keyword density map (2003–2024).

Figure 4 displays a density map generated using VOSviewer, which visualizes the co-occurrence frequency and relevance of the most representative keywords in research on OIUIC. This visualization facilitates the identification of the core concepts that structure the field, as well as those occupying peripheral or emerging positions. The brightest zones—depicted in shades of yellow and vivid green—indicate areas of high semantic density, highlighting keywords with strong frequency and connectivity. Notable among them are technology transfer, academic entrepreneurship, entrepreneurial university, UIKT, and commercialization, which constitute the most consolidated conceptual core in the analyzed literature. These terms reflect not only the maturity of the classical approach centered on technology transfer, but also its increasing articulation with entrepreneurial dynamics within universities. Together, this thematic nucleus acts as the structural foundation of the field, from which other lines of inquiry related to open innovation processes in inter-institutional settings emerge.

In the intermediate-density zones of the map, represented by lighter shades of green, lie terms that exhibit moderate frequency and a medium level of connectivity. Keywords such as knowledge management, absorptive capacity, collaborative innovation, networks, triple helix, and social capital represent research strands that, while not central to the field, are conceptually linked to the dynamics of open innovation in university–industry collaborations. These concepts contribute organizational, relational, and systemic perspectives that broaden the analytical understanding of OIUIC. Their position on the map suggests they form subfields in the process of consolidation, developed with less intensity or continuity than the core. These intermediate areas indicate the existence of thematic agendas that intersect with the conceptual nucleus and provide fertile ground for future research, particularly in relation to knowledge management, multi-actor collaboration, and regional innovation systems in the context of OIUIC.

The peripheral areas of the map cluster terms with low co-occurrence frequency and weaker thematic connectivity, including developing countries, entrepreneurial orientation, academic engagement, resource-based view, science parks, and student entrepreneurship. These keywords represent emerging lines within OIUIC research, often driven by specific contexts or academic communities exploring alternative agendas. Their scattered appearance suggests that these topics have not yet been fully integrated into the field's conceptual core, although they demonstrate growing thematic expansion. These emergent lines open opportunities for incorporating new perspectives, such as the territorialization of innovation, institutional capabilities in developing economies, or entrepreneurial orientation in university education. Collectively, the peripheral positioning of these concepts signals a diversification process that broadens the analytical scope of the field. This openness to less explored approaches enhances the intellectual vitality of the academic debate on open innovation in university–industry collaborations.

### 4.2 Most frequently cited articles

Table 3 presents the 10 most highly cited articles. The data indicate that the article titled “In search of complementarity in innovation strategy: Internal R&D and external knowledge acquisition” is the most frequently cited within the field of OIUIC, with a total of 1,878 citations. This information is particularly valuable for researchers and readers seeking to identify the most influential contributions in this area of study.

**Table 3:** Top ten most-cited publications in the corpus.

**Document title**	**Authors**	**Source**	**Citations**	**Overview**
In search of complementarity in innovation strategy: internal R&D and external knowledge acquisition	Cassiman and Veugelers (2006)	Management science	1,878	Assesses whether internal R&D and external knowledge acquisition complement each other in firms' innovation strategy using Belgian CIS data, showing that firms that combine make and buy achieve higher innovation performance and that complementarity depends on basic research intensity and appropriability conditions. The paper shifts the focus from whether complementarity exists to when and why it occurs, thereby informing the design of balanced search strategies.
Academic engagement and commercialization: a review of the literature on university-industry relations	Perkmann et al. (2013)	Research policy	1,683	Reviews university–industry relations by distinguishing academic engagement (collaborative research, contract research, consulting, informal advice) from commercialization (IP, licensing, spin-offs). Synthesizes evidence on antecedents and consequences at individual, organizational, and institutional levels, showing that engagement is more widespread, closely aligned with traditional research, and often pursued to access resources and learning, whereas commercialization follows different drivers and incentives. Highlights mixed impacts on productivity and secrecy, clarifies where organizational supports matter, and proposes a framework and research agenda to improve measures, comparability, and policy design.
University entrepreneurship: a taxonomy of the literature	Rothaermel et al. (2007)	Industrial and corporate change	1,108	Synthesizes 173 refereed articles on university entrepreneurship to map a fragmented field and propose a unifying framework. Identifies four streams that organize the literature: the entrepreneurial research university, productivity of technology transfer offices, new firm creation, and the environmental context including innovation networks. Documents a sharp rise in output after policy shifts such as Bayh Dole and shows publication concentration in a few journals, with many studies remaining a theoretical or descriptive. Reviews methods and units of analysis, noting a mix of qualitative case work and quantitative studies, and highlights gaps that motivate a future research agenda.
University-industry linkages in the UK: what are the factors underlying the variety of interactions with industry?	D'Este and Patel (2007)	Research policy	1,037	Analyses how UK academics interact with industry across a broad set of channels and what drives that variety. Using a large-scale survey of EPSRC funded researchers, the study shows that academics engage more often in consultancy, contract research, joint research, training, and meetings than in patenting or spin offs. Variation in engagement is shaped more by individual characteristics than by departmental or university level traits, though higher departmental industry income is associated with greater variety. The authors argue that policy should look beyond IPR commercialization to support a wider portfolio of knowledge transfer mechanisms, and the integration skills needed to bridge research and application.
University-industry relationships and open innovation: toward a research agenda	Perkmann and Walsh (2007)	International journal of management reviews	1,029	Synthesizes evidence on university–industry relationships such as collaborative research, research centers, contract research, and consulting, and distinguishes these channels from intellectual property transfer and human mobility. Shows that relationship-based mechanisms are widespread and often more consequential for innovation than licensing, supporting a non-linear, networked view of knowledge production. Maps when and why firms engage universities, highlights sectoral differences in preferred channels, and notes that the organization and management of partnerships remain under researched. Sets a research agenda on search and matching processes, partnership governance, and better indicators to assess outcomes and policy.
Research groups as “quasi-firms”: the invention of the entrepreneurial university	Etzkowitz (2003)	Research policy	985	Argues that the entrepreneurial university emerged from an inner academic logic and not only external pressures, with research groups functioning as quasi firms under competitive funding. Traces two academic revolutions from teaching to research and then to economic and social development, illustrated by Stanford's transition and its interface mechanisms such as technology transfer offices, incubators, and research centers. Develops models of separation vs. integration to manage conflicts of interest, and frames university–industry–government interactions within the triple helix as drivers of innovation and firm creation.
Investigating the factors that diminish the barriers to university-industry collaboration	Bruneel et al. (2010)	Research policy	940	Identifies which factors lower firms' perceived barriers to collaborating with universities, distinguishing orientation related obstacles from transaction related ones. Using a large survey of UK firms engaged in EPSRC funded projects and linked records of prior collaborations, the study finds that prior collaborative experience reduces orientation related barriers, while interorganizational trust lowers both orientation and transaction related barriers. Broader engagement across multiple interaction channels decreases orientation related barriers but increases transaction related barriers, reflecting added administrative and IP negotiations. Policy implications include fostering trust-based relationships and simple, ex ante IP arrangements, and recognizing that expanding channels of interaction can simultaneously ease cultural misalignment and raise transactional complexity.
The impact of network capabilities and entrepreneurial orientation on university spin-off performance	Walter et al. (2006)	Journal of business venturing	869	Examines how network capability and entrepreneurial orientation shape the performance of university spin offs using a dataset of 149 firms. Finds that network capability positively relates to sales growth, sales per employee, profit attainment, customer relationship quality, competitive advantages, and long-term survival. Entrepreneurial orientation does not directly raise financial outcomes but is associated with competitive advantages and better customer relationships. Network capability strengthens the link between entrepreneurial orientation and performance, suggesting that entrepreneurial drive delivers results when firms can develop and use partnerships through coordination, relational skills, partner knowledge, and internal communication.
Critical junctures in the development of university high-tech spinout companies	Vohora et al. (2004)	Research policy	792	Multiple case study of nine UK university high tech spinouts that traces venture development across five phases: research, opportunity framing, pre organization, re-orientation, and sustainable returns. Identifies four critical junctures that ventures must cross to progress: opportunity recognition, entrepreneurial commitment, venture credibility, and venture sustainability. Shows how resource gaps, capability building, and social capital shape whether teams navigate these junctures, with the locus of entrepreneurship shifting from the founding academic to a broader team as the firm grows. Frames dynamic capability development as central to overcoming bottlenecks and achieving durable performance.
Entrepreneurial orientation, technology transfer and spinoff performance of U.S. universities	O'Shea et al. (2005)	Research policy	732	Examines why some universities generate more technology-based spin offs by applying a resource-based view with panel data on 141 US institutions from 1980 to 2001. Tests eight hypotheses linking institutional history, faculty quality, science and engineering funding composition, industry support, technology transfer office staffing, and incubator presence to spin off counts using random effects negative binomial models. Finds strong path dependence, positive effects of faculty quality, federal funding scale and orientation toward life sciences, chemistry and computer science, higher shares of industry funding, and larger TTO staffing, while incubator presence is not significant. Concludes that combinations of human, financial, and commercial resources explain inter university variation in spin off activity and offers policy implications for building capability, incentives, and partnerships.

**Figure 5:**
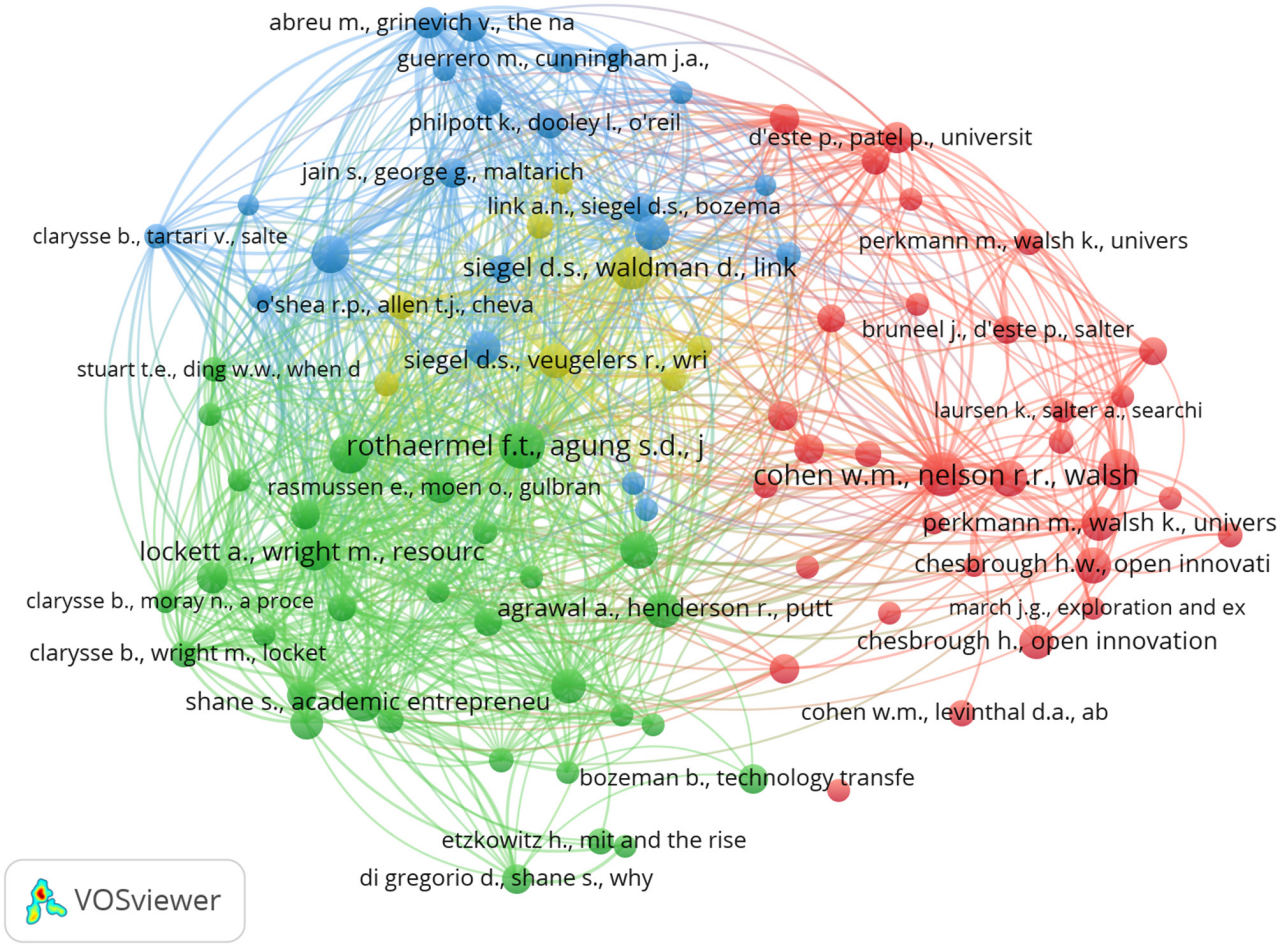
Co-citation network (2003-2024).

To advance the understanding of the conceptual structure of research on OIUIC, a complementary co-citation analysis was performed. Whereas, co-word analysis delineates the thematic composition of the field by capturing keyword associations, co-citation analysis uncovers its intellectual foundations by examining how frequently pairs of authors or documents are cited together in subsequent publications (Jeong et al., 2014). Figure 5 presents a co-citation network comprising 93 highly cited references—each cited at least 35 times—grouped into four coherent clusters. These clusters reflect distinct schools of thought that have shaped the intellectual evolution of the field. Their dense interconnections point to a mature and well-integrated research domain. This analysis extends the insights of the co-word mapping by highlighting the foundational contributions that structure and legitimize the emergence of major thematic trajectories (Lafia et al., 2021). Together, these methods provide a comprehensive depiction of the field's conceptual architecture and illuminate how seminal works have influenced the development of theoretical perspectives and scholarly debates in OIUIC research.

Cluster 1 (red) is the most extensive and conceptually central, composed of 32 documents anchored in the influential work of Cohen, Nelson, and Walsh. These studies emphasize the concept of absorptive capacity and its critical role in facilitating knowledge transfer within university settings. The cluster highlights how organizational routines, search processes, and internal capabilities shape the ability of academic institutions to engage with external knowledge environments. Cluster 2 (green) also comprises 32 documents and is structured around *Shane, Wright, Clarysse*, and *Rothaermel*. This group focuses on academic entrepreneurship, university spin-offs, and the mechanisms of technology commercialization, positioning universities as entrepreneurial actors. Together, these clusters represent two dominant intellectual currents in the field. Cluster 3 (blue), with 21 documents, centers on the contributions of Siegel, Link, and Bozeman, who examine university–industry collaboration, research evaluation, and institutional performance from policy and governance perspectives.

Cluster 4 (yellow) contains eight documents and captures a more emergent stream of research focused on institutional and economic dimensions of innovation. Anchored in the work of Waldman, Veugelers, and others, this cluster explores how public policy, performance measurement, and academic incentives influence universities' roles in innovation systems. While smaller in size, it plays a complementary role by highlighting contextual and structural conditions that enable or constrain knowledge exchange. The overall co-citation network shows a well-integrated conceptual landscape, in which foundational references are not isolated but interlinked across themes. This confirms that research on OIUIC has developed around interconnected theoretical pillars—technology transfer, absorptive capacity, academic entrepreneurship, and university–industry engagement. By combining co-word and co-citation analyses, this study provides a robust and multi-dimensional perspective on the conceptual structure of the field, aligning with the goal of capturing its intellectual foundations and thematic evolution.

### 4.3 Top authors

Table 4 presents the ten most prolific authors in the field of open innovation in university–industry collaborations (OIUIC). According to the data, Mike T. Wright stands out as the leading scholar in terms of publication output, with a total of 30 published documents. This type of information is useful for identifying the most influential contributors shaping the field, as well as for recognizing key figures whose work has guided both the theoretical and empirical development of the specialized literature.

**Table 4:** Top authors.

**Author**	**University**	**Country**	**Documents**
Mike T. Wright	Imperial College London	United Kingdom	30
Einar Rasmussen	Nord University	Norway	21
David B. Audretsch	Indiana University Bloomington	United States	18
Rosa Grimaldi	University of Bologna	Italy	18
Giustina Secundo	LUM Giuseppe Degennaro University	Italy	18
James A. Cunningham	Lund University	Sweden	16
Maribel Guerrero	Arizona State University	United States	16
Christopher S. Hayter	Georgia Institute of Technology	United States	15
Albert N. Link	The University of North Carolina at Greensboro	United States	15
Donald S. Siegel	Arizona State University, Downtown Phoenix campus	United States	15

**Figure 6:**
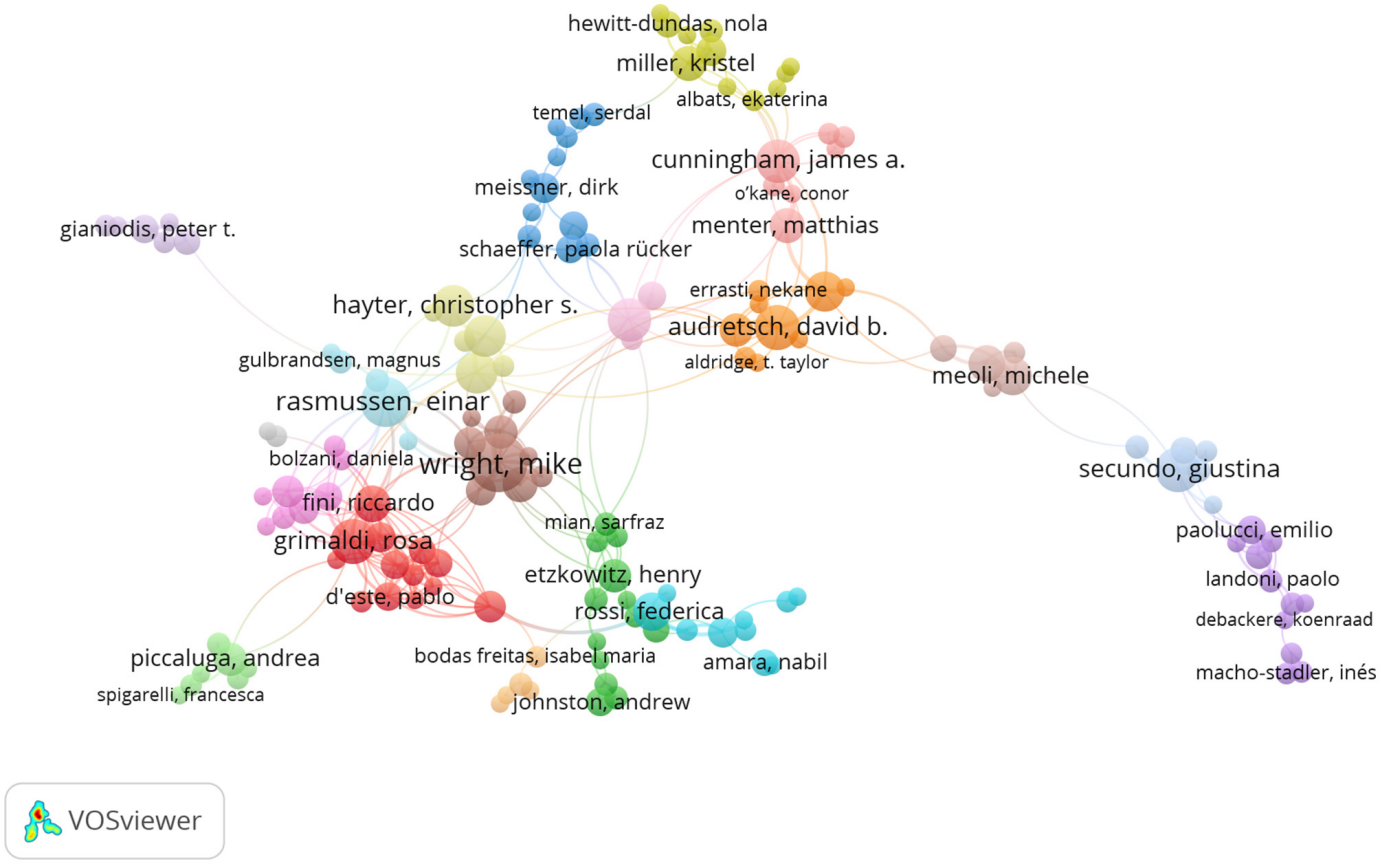
Co-authorship network (2003–2024).

Figure 6 presents a co-authorship analysis that complements the co-word mapping by illustrating how patterns of disciplinary mixing within collaborative networks influence knowledge diffusion and the formation of interdisciplinary research communities (Feng and Kirkley, 2020). The network was constructed using a minimum threshold of three co-authored documents and ten citations per author, resulting in the inclusion of 330 highly productive and influential scholars. Prominent nodes—such as Mike Wright, Einar Rasmussen, and David B. Audretsch—emerge due to their high frequency of co-authorship and strong network centrality, underscoring their pivotal role in shaping both theoretical frameworks and empirical agendas. The visualization reveals clearly delineated clusters, each represented by distinct colors, which correspond to research communities with shared intellectual trajectories. The proximity of certain clusters suggests robust interdisciplinary collaboration, whereas greater distances indicate weaker ties across research streams. This network adds a relational layer to the field's conceptual analysis, demonstrating how collaborative structures support and reflect the intellectual architecture of OIUIC research.

The network reveals varying levels of integration among authors contributing to the development of OIUIC scholarship. Some clusters exhibit dense and cohesive structures, reflecting well-established communities with high levels of collaborative activity. In contrast, other groups are more loosely connected, which may reflect thematic specialization, institutional separation, or geographic fragmentation. For example, the group led by Giustina Secundo and Emilio Paolucci appears in a peripheral area of the network, suggesting limited integration with the more consolidated co-authorship cores. Similarly, Peter T. Gianiodis emerges as an isolated node, indicating low connectivity within the overall network structure. These structural differences help identify both consolidated research hubs and areas with potential for further integration. Understanding these collaborative configurations is essential to contextualize how conceptual frameworks in the field are constructed, disseminated, and transformed over time.

The co-authorship network offers a relational perspective on the intellectual evolution of OIUIC research, complementing the thematic insights derived from co-word analysis. By identifying central authors, active clusters, and peripheral zones, the map captures the dynamics of academic production that sustain the field's conceptual development. The interconnectivity across scientific communities points to the presence of bridging scholars who facilitate knowledge exchange between different perspectives, thereby contributing to the field's consolidation and expansion. At the same time, the fragmentation of certain groups suggests opportunities to enhance interdisciplinary connections and address thematic silos. This analysis not only helps identify key contributors but also visualizes the collaborative trajectories that shape the main theoretical and empirical currents in OIUIC research. Overall, the co-authorship network reinforces the analytical scope of this study by demonstrating how researcher relationships co-evolve with the concepts, topics, and frameworks that define the field.

### 4.4 Top countries

Table 5 presents the distribution of scientific output in the field of open innovation in university–industry collaborations (OIUIC) across the ten most productive countries. The United Kingdom and the United States lead the ranking, each contributing 469 publications. Together, they account for a significant portion of the overall output, indicating their dominant position in shaping the research agenda. Italy follows with 323 documents, while Spain and Germany report comparable figures, with 182 and 179 publications, respectively. China ranks just behind, with 170 documents, reflecting its growing engagement with the topic. The Netherlands, France, and Australia present more moderate contributions, with totals ranging from 103 to 122 publications. These patterns suggest that OIUIC research is strongly concentrated in a small group of countries, particularly in Europe and North America. The data also highlight the prominent role of national research systems in supporting high impact scholarship and point to the importance of sustained institutional capacity in maintaining academic visibility.

**Table 5:** Top countries.

**Country**	**Number of documents**
United Kingdom	469
United States	469
Italy	323
Spain	182
Germany	179
China	170
Netherlands	122
France	110
Australia	103
Brazil	96

At the lower end of the ranking, Brazil appears with 96 publications, representing a modest but visible contribution to the field. This positioning illustrates a broader pattern of geographic concentration in OIUIC research, with leading countries significantly outpacing others in terms of publication volume. The gap between the most and least productive nations within this group underscores persistent inequalities in global knowledge production. Such disparities may reflect differences in research funding, institutional infrastructure, or international collaboration networks. Notably, the strong presence of European countries within the top ten indicates a regionally embedded academic interest in university–industry relations and open innovation models. Meanwhile, the relatively limited participation of countries outside Europe and North America suggests potential areas for expansion. Strengthening global collaboration, fostering capacity building in underrepresented regions, and promoting inclusive research agendas could help diversify the field. This distribution ultimately reinforces the need for more balanced academic participation to enhance the global relevance and reach of OIUIC scholarship.

**Figure 7:**
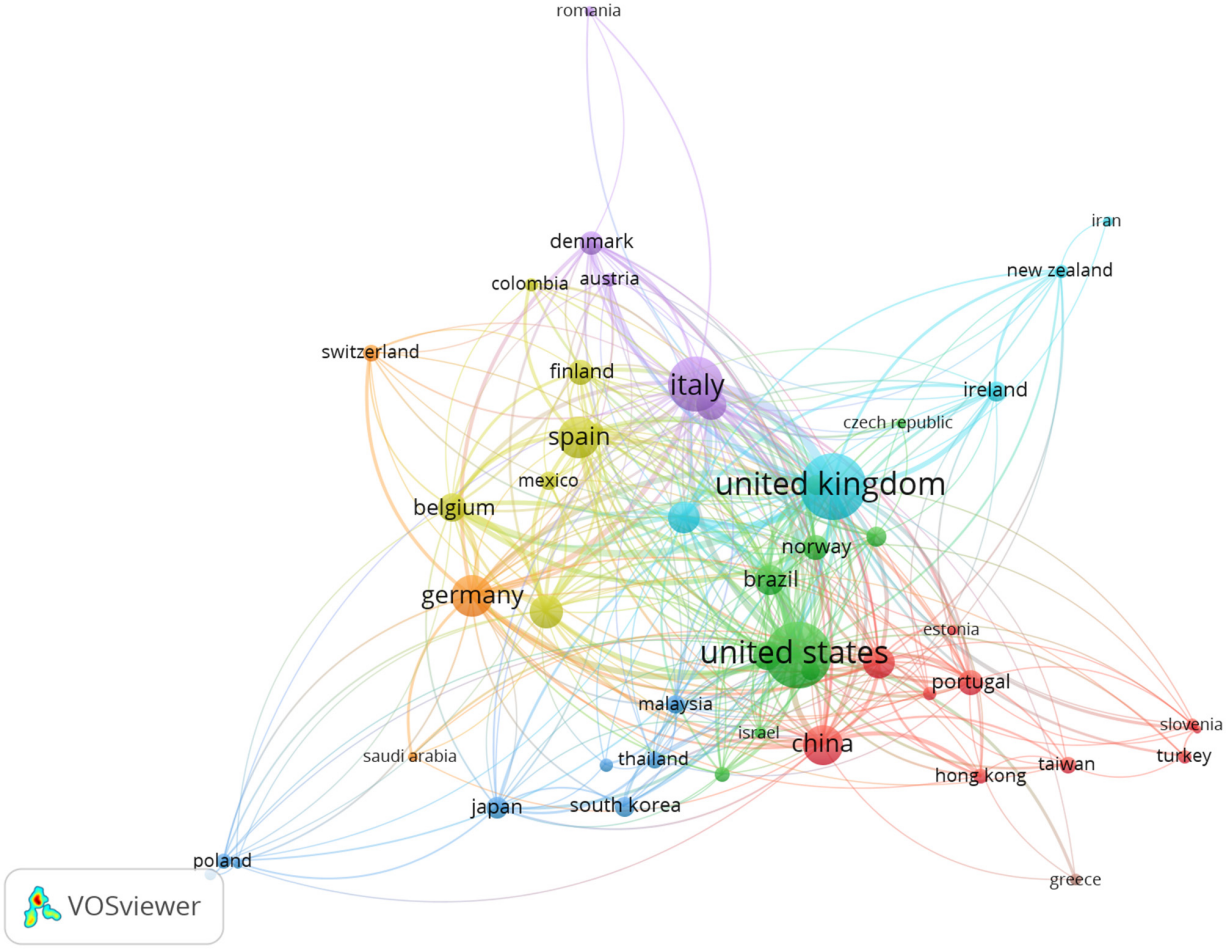
Co-authorship network of countries (2003–2024).

Figure 7 presents the country-level co-authorship network, illustrating the structure of international collaboration in research on open innovation in university–industry collaborations (OIUIC). Of the 211 countries identified in the database, 49 met the minimum threshold of 10 documents and 20 citations, and were therefore included in the analysis. Node size reflects publication volume, while link thickness indicates the intensity of co-authorship relations. The United States, United Kingdom, China, Italy, and Germany emerge as central hubs of scientific production, exhibiting high connectivity across the network. These countries function as key knowledge brokers, facilitating collaborative flows across multiple regions. The map also reveals the presence of distinct regional clusters, where countries tend to group based on shared geographic or linguistic ties. This structure offers a comprehensive view of how scientific alliances are globally distributed and highlights the countries that lead the consolidation of the international research landscape in OIUIC.

The network reveals a collaborative dynamic in which countries with high publication output—such as the United States and United Kingdom—not only occupy central positions but also maintain linkages with diverse regions, thereby extending the reach of their academic networks. The United Kingdom maintains strong ties with Ireland, Australia, New Zealand, and the Netherlands, while the United States is closely connected to China, Brazil, Germany, and Malaysia. In parallel, Italy and Spain are embedded within a dense European cluster that includes France, Switzerland, Belgium, and Finland. Countries with lower publication volumes, such as Norway, Mexico, Austria, and Colombia, appear integrated into these networks, suggesting active internationalization processes. While their quantitative participation is limited, their location within central clusters reflects a stable linkage with major academic hubs. These collaborative patterns highlight both the concentration of scientific production and a growing geographic openness that contributes to the expansion and diversification of the field.

At the same time, the visualization highlights peripheral zones composed of countries with lower levels of international co-authorship, such as Iran, Poland, Turkey, Romania, Slovenia, and Greece. Although positioned at the margins of the network, their presence signals a gradual integration into the global academic ecosystem. Despite their more limited connectivity, their participation reflects a trend toward international openness and the development of strategic alliances in emerging contexts. This progressive expansion of academic collaboration helps democratize access to scientific production and fosters the inclusion of new territorial perspectives. Overall, the country-level co-authorship network reveals a dual dynamic: on the one hand, a concentration of scientific output among a consolidated core of highly connected countries; on the other, the gradual incorporation of underrepresented regions that enhance the diversity, complexity, and global reach of OIUIC research.

### 4.5 Top journals

Table 6 summarizes the 10 most highly cited journals contributing to the field of open innovation in university–industry collaborations (OIUIC). These journals represent key venues for the dissemination of research on innovation management, technology transfer, and academic–industry engagement. *Research Policy* clearly dominates the ranking, with over 465,000 citations and a high h-index, underscoring its central role in shaping the field's intellectual agenda. *Technological Forecasting and Social Change* and *Technovation* follow in prominence, both featuring strong impact factors and a focus on strategy, innovation systems, and policy. Other journals such as *The Journal of Technology Transfer* and *IEEE Transactions on Engineering Management* also play a critical role in advancing research at the intersection of organizational studies and innovation policy. The list reflects both disciplinary diversity and geographical concentration, with the United States and the United Kingdom hosting most of these outlets. The inclusion of *Industry and Higher Education* and *The Journal of Technology Management and Innovation* reflects a growing interest in applied and regionally grounded perspectives within the field.

**Table 6:** Top journals.

**Journal**	**Scope**	**Country**	**Publisher**	***H*-Index (2024)**	**Impact factor (2024)**	**Citations (2003–2024)**	**Documents**
Research policy	Business, management and accounting; decision sciences; engineering	Netherlands	Elsevier	289	7.5	465,647	160
Technological forecasting and social change	Applied psychology; management science and operations research; business and international management; management of technology and innovation; strategy and management	United States	Elsevier	179	12.9	335,067	97
Technovation	Management science and operations research; business and international management; management of technology and innovation; strategy and management	United Kingdom	Elsevier	159	11.1	138,538	85
IEEE transactions on engineering management	Business, management and accounting; strategy and management; engineering; electrical and electronic engineering	United States	Institute of Electrical and Electronics Engineers (IEEE)	112	4.6	94,015	40
Journal of technology transfer	Business, management and accounting; strategy and management; engineering; engineering (miscellaneous)	United States	Kluwer Academic Publishers	102	5.4	55,596	359
Technology analysis and strategic management	Business, management and accounting; strategy and management; decision sciences; management science and operations research	United Kingdom	Routledge	84	3.74	53,882	47
International journal of technology management	Business, management and accounting; industrial relations; strategy and management; computer science applications; engineering (miscellaneous); social sciences; law	United Kingdom	Inderscience Enterprises Ltd	66	1.53	35,604	43
Industry and innovation	Business, management and accounting (miscellaneous); management of technology and innovation	United Kingdom	Routledge	72	3.4	30,725	33
Industry and higher education	Business, management and accounting; business and international management; social sciences; education	United States	SAGE Publications	32	2.91	10,368	146
Journal of technology management and innovation	Business, management and accounting; management of technology and innovation	Chile	Universidad Alberto Hurtado	35	1.28	8,945	39

**Figure 8:**
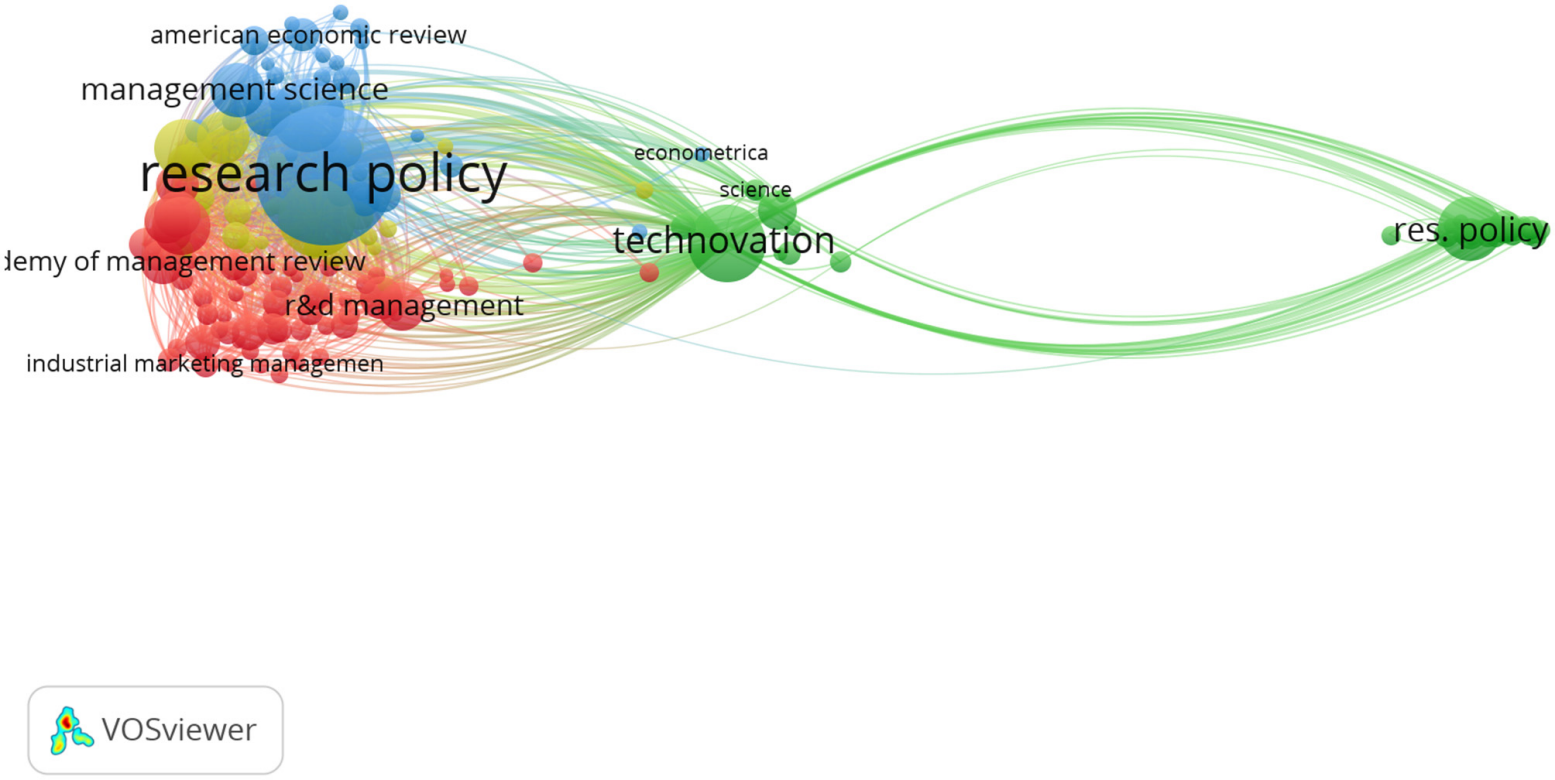
Co-citation network of journals (2003–2024).

The journal co-citation analysis complements the co-word approach by offering an additional perspective on the conceptual structure of research on open innovation in university–industry collaborations (OIUIC). Based on a total of 151 sources, a network was constructed using co-citation link strength to identify the most influential academic journals and their degree of thematic alignment. The resulting visualization, organized into four color-coded clusters, groups journals with similar research orientations, reflecting the intellectual configuration of the field. A total of 18,975 connections were identified, with a cumulative link strength of 4,301,902, indicating a high level of bibliographic interconnectivity. Within this network, *Research Policy, Technovation*, and *Management Science* emerge as central nodes, underscoring their structuring role in consolidating interdisciplinary knowledge on open innovation and technology transfer in academic and industrial contexts. Figure 8 displays the co-citation network of journals covering the period 2003–2024.

The first cluster (red) consists of journals focused on organizational management, innovation, and strategic behavior. Prominent nodes include the *Academy of Management Review, Journal of Management, Industrial Marketing Management*, and *R&D Management*. These publications address key issues in innovation management, organizational design, and collaborative governance, positioning this cluster as a central reference for studies on internal dynamics within organizations engaged in open innovation processes. The second cluster (green), structured around *Research Policy* and *Technovation*, brings together journals specializing in innovation policy, knowledge transfer, and university–industry collaboration. Their central position in the network reflects the conceptual importance of these themes in the OIUIC field and highlights their role in bridging macro-level policy analysis with micro-level organizational practices.

The third cluster (blue) includes journals with a more foundational and quantitative orientation, such as *American Economic Review, Econometrica, Science*, and *Management Science*. This group represents the field's theoretical-economic dimension, addressing models of efficiency, organizational rationality, and market dynamics from a formal analytical perspective. Its connections with clusters focused on management and technology transfer suggest that economic theory remains a key conceptual underpinning for the development of OIUIC research. Finally, the fourth cluster (yellow) includes journals such as *Education* + *Training* and *Economic Development Quarterly*, which incorporate educational and territorial dimensions into the study of innovation. These outlets emphasize the role of human capital, professional training, and regional development in shaping collaborative ecosystems. Taken together, the four clusters illustrate the convergence of multiple disciplinary traditions around the analysis of open innovation in university–industry environments.

### 4.6 Top universities

Table 7 presents the ten universities with the highest number of publications in the field of open innovation in university–industry collaborations (OIUIC), based on the data analyzed. This type of information is valuable for identifying academic institutions with a strong track record in scientific production on the topic, and it may help guide the selection of strategic partners for future international collaborations. The analysis reveals that a total of 3,105 universities and research institutes have contributed to the field, with the University of Bologna ranking first, with 40 published documents. It is followed by Imperial College Business School (United Kingdom), KU Leuven, and Ghent University (both from Belgium), each with more than 30 publications. The predominance of European universities in the ranking highlights the region's institutional commitment to advancing knowledge on open innovation. This distribution also suggests the existence of consolidated academic networks in Europe that are leading scientific efforts to strengthen university–industry linkages within collaborative innovation contexts.

**Table 7:** Top universities.

**University**	**Country**	**Documents**
University of Bologna	Italy	40
Imperial College Business School	United Kingdom	34
KU Leuven	Belgium	32
Ghent University	Belgium	32
University of Turin	Italy	31
Lund University	Sweden	29
HSE University	Russia	27
Polytechnic University of Valencia	Spain	25
University of Nottingham	United Kingdom	25
University of Naples Federico II	Italy	25

**Figure 9:**
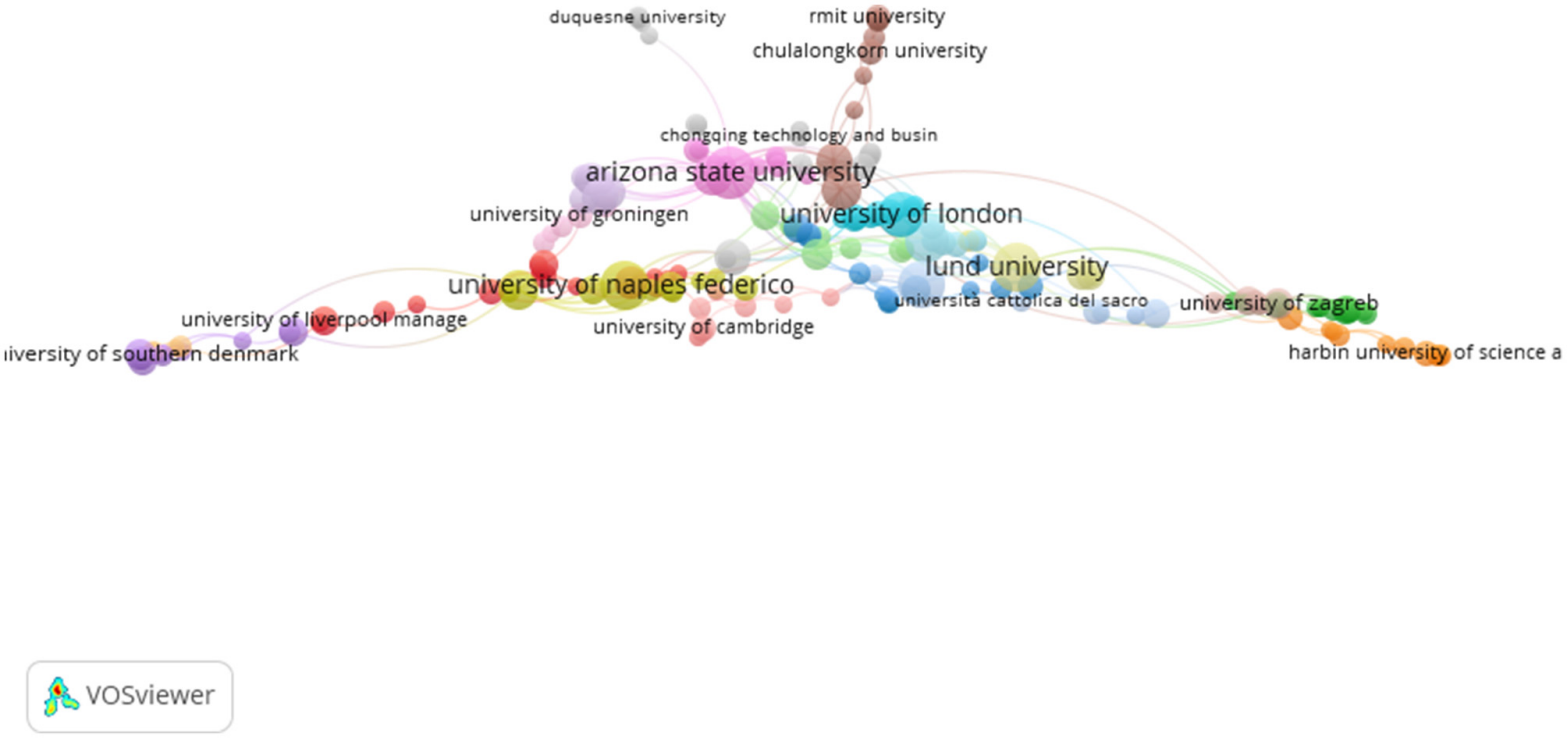
Co-authorship network between universities (2003–2024).

Figure 9 illustrates the university co-authorship network, constructed from a dataset of 4,251 organizations involved in academic publishing within the field under study. Applying a minimum threshold of three documents per institution, the analysis focused on a subset of 177 universities and research centers. These entities, represented as nodes in the network visualization, reflect the structure of inter-institutional collaboration. Node size corresponds to the volume of publications, while link thickness indicates the frequency of bilateral co-authorship. This approach enables the identification of institutional participation levels, as well as the presence of cohesive clusters that span across national and regional boundaries, highlighting differentiated patterns of scientific cooperation.

Institutions with the highest total link strength include Arizona State University (21), Universidad del Desarrollo (17), University of Naples Federico II, and University of Campinas (13 each), signaling a strong engagement in collaborative research. Other notable nodes include Lund University, the National Research University Higher School of Economics, and the University of Salento, suggesting a geographically diverse network topology. The coexistence of well-established universities with emerging institutions reflects a distributed configuration of global scientific collaboration. These findings point to the presence of strategic nodes and meaningful inter-organizational relationships that may inform future academic partnerships and support evidence-based internationalization policies.

## 5 Discussion and conclusions

This study has examined the evolution, structure, and emerging directions of research on Open Innovation in University–Industry Collaborations (OIUIC) between 2003 and 2024. Drawing upon co-word analysis and social network mapping, it has provided a comprehensive overview of the field's conceptual architecture, thematic development, and scholarly impact. The methodological approach enabled the identification of key intellectual clusters, the differentiation between mature and emerging research areas, and the tracing of shifts in conceptual focus over two decades. Moreover, the study has highlighted influential authors, institutions, and journals that have significantly shaped academic production in this domain. Collectively, these insights contribute to a deeper understanding of how OIUIC research has evolved in response to global challenges such as sustainability and institutional transformation. The findings are particularly relevant to researchers, policymakers, and practitioners engaged in designing inclusive, adaptive, and dynamic innovation ecosystems capable of addressing complex socio-technical demands.

The first *research question* examined the conceptual structure underpinning OIUIC research. The co-word analysis revealed that the field is anchored in five dominant conceptual clusters: technology transfer, university–industry knowledge transfer (UIKT), academic entrepreneurship, knowledge exchange, and the university itself. These clusters represent both formal mechanisms of commercialization and broader collaborative processes rooted in institutional partnerships. Related terms such as absorptive capacity, entrepreneurial university, and collaborative innovation emphasized the role of mutual learning and co-creation within open innovation settings. Spatial and systemic constructs—including triple helix, science parks, and regional innovation systems—further illustrated the embeddedness of OIUIC within territorial development strategies and innovation ecosystems. Additionally, the presence of terms like developing countries and entrepreneurial orientation signaled growing attention to institutional diversity, contextual adaptation, and inclusivity. Together, these findings confirm the multidimensional character of OIUIC as a research domain shaped by academic, organizational, and policy-driven influences across multiple spatial and institutional scales.

The second *research question* examines how the field's themes evolve over time. The results point to a diversification of research agendas, distinguishing among consolidated, mature, declining, and emerging areas. Technology transfer, university–industry knowledge transfer, and academic entrepreneurship remain foundational concepts across the period, sustaining their salience as new priorities appear. By contrast, triple helix and entrepreneurship education show signs of maturity rather than saturation, with limited recent extension of their theoretical scope. Specialized but less interconnected topics such as open innovation ecosystems and higher education institutions have gained visibility, although they remain under integrated within dominant clusters. We also observe declining frequencies for keywords such as university patents and licensing, suggesting a shift away from intellectual property centered approaches. Emerging themes including digital transformation, sustainability, and the role of developing countries reflect growing attention to global challenges and institutional adaptation. Taken together, this dynamic evolution underscores the bridging role of UIKT between mature research areas and newer lines of inquiry.

The *third research* question investigated the temporal development of OIUIC literature across two decades. The analysis showed that during the early 2000s, the field was predominantly shaped by concerns with formal knowledge appropriation and commercialization—reflected in the prevalence of terms such as technology transfer, university patents, and licensing. Over time, relational and institutional perspectives gained traction, exemplified by the rise of academic entrepreneurship, trust, absorptive capacity, and collaborative governance. More recent literature has emphasized co-creation, value generation, and distributed innovation dynamics, integrating keywords like knowledge creation, gamification, and institutional learning. This shift reflects a transition from linear models of innovation to more networked, multi-actor configurations embedded in territorial and socio-political contexts. The evolution of themes indicates not only conceptual maturity but also alignment with broader agendas such as sustainable development and digital transformation. This thematic progression has contributed to reframing OIUIC as a driver of systemic change and knowledge democratization.

The *fourth research question* identified the most influential actors and contributions within the OIUIC knowledge domain. The most cited article was “In search of complementarity in innovation strategy,” with 1,878 citations, illustrating its foundational role in shaping research on internal and external knowledge integration. Mike T. Wright, Einar Rasmussen, and David B. Audretsch emerged as leading authors based on publication output and citation impact. Institutionally, the University of Bologna stood out for its productivity, while the United Kingdom and the United States were the top contributing countries in terms of volume. *Research Policy* was the most cited journal, confirming its intellectual centrality in the field, while *The Journal of Technology Transfer* was the most prolific outlet for OIUIC-related publications. These patterns highlight the existence of a dense academic network that has both structured and advanced the field. They also provide a foundation for future collaboration and benchmarking, particularly in identifying opportunities for cross-institutional partnerships and inter-regional knowledge exchange.

The fifth *research question* focused on identifying promising directions for future inquiry. The findings suggest the need to broaden existing analytical frameworks by incorporating dimensions such as territorial equity, environmental sustainability, and social engagement into the study of OIUIC. Future research should examine entrepreneurial orientation in developing economies, institutional capacity building in resource-constrained settings, and governance models that support distributed innovation. Additional attention should be paid to the role of intermediary actors, regional innovation networks, and inter-organizational learning mechanisms that foster co-creation and systemic resilience. Furthermore, emerging digital tools and collaborative platforms open new avenues for exploring innovation beyond traditional institutional boundaries and across geographical regions. These priorities call for more integrative theoretical approaches and mixed-method research designs capable of capturing the nuanced dynamics of university–industry–society interactions. Emphasizing inclusivity, adaptability, and context sensitivity, this forward-looking agenda aligns with global policy trends and reinforces the relevance of OIUIC research in addressing current and future societal challenges.

Our findings suggest both divergence and common ground between the Global North and the Global South in OIUIC. Northern systems typically show denser collaboration networks, stronger brokerage positions, and greater emphasis on technology transfer, patenting, and commercialization, while many Southern contexts display thinner ties, higher dependence on external funding, and the prominence of engagement, capability building, and inclusive innovation. Yet several mechanisms operate across regions. University–industry knowledge transfer remains the key bridge connecting research, entrepreneurship, and policy. Effective TTO routines, relational capital with firms, and absorptive capacity in partner organizations consistently support outcomes. Governance that aligns incentives for academics and practitioners, transparent contracting, and predictable evaluation criteria also travels well. Policy mixes should therefore adapt the weight of instruments rather than their identity. Northern contexts benefit from tools that reduce transaction costs and scale successful partnerships. Southern contexts gain from long term alliances, TTO capacity development, context sensitive IP templates, and infrastructure that strengthens networks without crowding out local priorities.

Beyond the core findings, we address structural barriers with concrete strategies. To reduce incentive misalignment, partners should agree in advance on shared objectives, milestones, and success metrics, and embed engagement outputs in academic workload and recognition systems. Trust deficits can be mitigated through repeated interactions, boundary spanners, and co located teams, starting with low complexity pilots and scaling as performance evidence accumulates. To navigate regulatory rigidity, universities and firms can adopt standard contracting playbooks that include model NDAs, master research agreements, and clear background and foreground IP clauses, alongside pre-approved templates for data governance and confidentiality. Divergent timeframes are best managed with stage gate governance, time boxed deliverables, and a portfolio that links long horizon projects to near term proofs of concept. Intermediary organizations such as technology transfer offices, living labs, and consortia should provide joint steering committees, dispute resolution routines, and transparent dashboards for monitoring KPIs. Together, these mechanisms translate intent into executable collaboration and increase the durability of OIUIC initiatives.

## 6 Research limits

This study has limitations that merit acknowledgment. Although we combine quantitative bibliometrics with qualitative synthesis, the qualitative layer relied on metadata such as titles, abstracts, and keywords rather than full text, which may constrain conceptual nuance and interpretive depth. Reliance on Scopus introduces a possible database effect because coverage expansion over time can affect estimates of trend magnitude, and the predominance of English language records may bias visibility toward Anglophone contexts while understating contributions from other regions. Dependence on a single index can also omit relevant publications in regional outlets or practitioner journals. Methodologically, results are sensitive to parameter choices, for example minimum keyword frequency, normalization method, network cutoffs, clustering resolution, and layout algorithm, as well as keyword harmonization and author or institution disambiguation. Finally, the 2003 to 2024 window may undercapture citation accrual for recent publications and may not fully reflect the delayed diffusion of OIUIC ideas across domains.

Future research should convert broad avenues into concrete, testable questions with clear constructs, units, and outcomes. First, does digital transformation moderate the effect of university–industry knowledge transfer on firms' product innovation, assessed through new product sales and co authored patents. Second, how do sustainability goals reweight universities' portfolios between commercialization and engagement, proxied by licensing revenue, spin offs, community partnerships, and carbon or circularity targets. Third, which technology transfer office practices reduce contracting frictions for small and medium enterprises, and with what effects on time to agreement and collaboration depth. Fourth, do inclusive innovation programmes in the Global South increase local firms' absorptive capacity and collaboration persistence relative to matched regions. Fifth, do bridging positions in the co word and co authorship networks predict subsequent cross sector projects and competitive funding wins. Addressing these questions will align measurement with mechanisms and provide cumulative evidence on when and why OIUIC arrangements deliver innovation, inclusion, and sustainability outcomes.

## Data Availability

The data analyzed in this study is subject to the following licenses/restrictions: The dataset was obtained from Scopus (Elsevier) and is subject to licensing restrictions. Raw data cannot be shared publicly but can be accessed through institutional or individual subscription. Requests to access these datasets should be directed to Elsevier – Scopus database website: https://www.scopus.com Support contact: https://service.elsevier.com.
